# Socio‐Economic Determinants and Regional Prevalence of Disorders of Gut‐Brain Interaction in the Netherlands: Results From the Rome Foundation Global Epidemiology Study

**DOI:** 10.1111/nmo.70181

**Published:** 2025-10-16

**Authors:** F. Veldman, F. Innocenti, M. A. Benninga, A. D. Sperber, O. S. Palsson, S. I. Bangdiwala, D. Keszthelyi

**Affiliations:** ^1^ Department of Gastroenterology‐Hepatology, NUTRIM Research Institute of Nutrition and Translational Research in Metabolism Maastricht University Medical Center Maastricht the Netherlands; ^2^ Department of Methodology and Statistics, Faculty of Health Medicine and Life Sciences, Care and Public Health Research Institute (CAPHRI) Maastricht University Maastricht the Netherlands; ^3^ Department of Pediatric Gastroenterology, Amsterdam Gastroenterology, Endocrinology, and Metabolism Research Institute, Amsterdam Reproduction and Development Research Institute, Emma Children's Hospital Amsterdam University Medical Center Amsterdam the Netherlands; ^4^ Faculty of Health Sciences Ben‐Gurion University of the Negev Be'er Sheva Israel; ^5^ Center for Functional GI & Motility Disorders University of North Carolina‐Chapel Hill Chapel Hill North Carolina USA; ^6^ Department of Health Research Methods, Evidence and Impact McMaster University Hamilton Ontario Canada; ^7^ Population Health Research Institute McMaster University Hamilton Ontario Canada

**Keywords:** disorders of gut‐brain interaction, epidemiology, Netherlands, Rome IV diagnostic criteria, socio‐economic factors

## Abstract

**Background:**

Disorders of gut‐brain interaction (DGBI) impose significant burdens worldwide, yet reliable regional prevalence estimates are lacking, limiting insight into potential geographic variation. Socio‐economic status (SES) may contribute but remains underexplored.

**Methods:**

Data were obtained from the Dutch cohort of the Rome Foundation Global Epidemiology Study, including the Rome IV Adult Diagnostic Questionnaire, sociodemographic items, and SES indicators. The Netherlands was divided into three regions (Region 1: South; Region 2: West; Region 3: North‐East). Regional prevalence was calculated for any DGBI, groups, and subtypes. Associations with SES were examined using logistic regression.

**Key Results:**

2008 participants (50% female; age: 48 years [IQR: 31]; BMI: 25.25 kg/m^2^ [IQR: 5.98]) were included. Overall DGBI prevalence was 30.63% (95% CI: 28.65–32.68), with no significant regional differences (Region 1: 30.40%, 95% CI [26.61–34.47]; Region 2: 30.19%, 95% CI [27.19–33.36]; Region 3: 31.45%, 95% CI [27.96–35.16]; *p* = 1.000). No regional variation was found across DGBI groups or subtypes (all *p* > 0.05). Younger age (OR = 0.98, 95% CI [0.98–0.99]), female sex (OR = 2.10, 95% CI [1.70–2.59]), underweight (OR = 2.53, 95% CI [1.36–4.73]), and obesity (OR = 1.59, 95% CI [1.19–2.13]) were associated with higher odds of DGBI. In models adjusted for age, sex, and BMI, limited/no healthcare access (OR = 2.47, 95% CI [1.62–3.77], *p* < 0.0001) was associated with higher DGBI odds, whereas employment showed lower odds (OR = 0.65, 95% CI [0.52–0.81], *p* < 0.001). Other SES indicators were not associated.

**Conclusion and Inferences:**

DGBI affect nearly one‐third of adults in the Netherlands, with no regional variation in prevalence. Putative risk factors include limited/no healthcare access and unemployment, supporting consideration of socioeconomic determinants in DGBI care, although further research is warranted.

## Graphical abstract

Disorders of gut‐brain interaction affect nearly one‐third of adults in the Netherlands, with no regional variation in prevalence. Putative risk factors include limited or no access to healthcare and unemployment, highlighting the importance of addressing specific socioeconomic determinants in recognition and management, although further research is warranted.

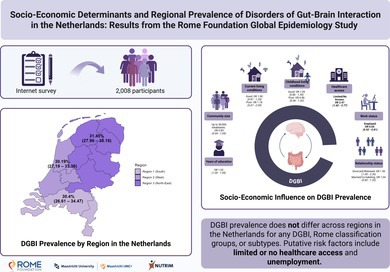

**Summary**

- DGBI prevalence does not differ across regions in the Netherlands for any DGBI, Rome classification groups, or subtypes, supporting nationwide rather than region‐specific strategies.
- Limited or no healthcare access and unemployment are associated with higher odds of DGBI, highlighting the importance of addressing specific socio‐economic determinants in recognition and management.
- Further research utilizing standardized SES indices is warranted to better understand the overall role of socioeconomic status to the burden of DGBI.

## 1 Introduction

Disorders of gut‐brain interaction (DGBI) are highly prevalent worldwide [1]. A considerable proportion of these individuals seek medical care either for bowel‐related issues (46.5%) or for any health concern (15.5%) once or more per month, contributing to a high patient load in both primary and gastroenterology care settings [1, 2, 3, 4].

The diagnosis of DGBI is primarily symptom‐based and relies on thorough history‐taking to identify characteristic symptom patterns [2]. Nonetheless, limited consultation time, along with insufficient awareness or expertise among healthcare providers, often lead to excessive testing, delayed diagnoses, and suboptimal treatment [2, 5]. This, in turn, contributes to increased healthcare utilization and a substantial symptom burden, affecting the physical, mental, and social aspects of patients' health‐related quality of life [1, 6]. Furthermore, high use of healthcare resources is associated with significant economic cost, including both direct medical expenses and productivity losses [2, 5, 7]. Together, these factors make DGBI a pressing and largely avoidable societal health challenge.

Differences between world regions, countries or regions within a single country may have important influences in this respect. In this study, we specifically focused on the Netherlands, being a highly developed country with notable regional and socio‐cultural differences [8]. Such variations may, in fact, be explained by cultural, ethnic, genetic, demographic (i.e., gender, age), environmental (i.e., dietary factors, living environment), and psychosocial (i.e., anxiety, depression, social cohesion) influences, as the distribution of DGBI is known to differ across populations [1, 4, 9]. Socio‐economic status (SES) has also been linked to DGBI, yet its role in prevalence remains insufficiently explored [9]. Existing evidence is limited and inconsistent [9, 10]. Drossman et al. [11] surveyed 5430 households in the United States and found that lower income was associated with a higher frequency and greater severity of DGBI symptoms. Similarly, Bytzer et al. [12] identified low socioeconomic class as a risk factor for both upper and lower gastrointestinal symptoms. Nevertheless, a meta‐analysis on 80 studies on IBS, only four of which examined SES, failed to confirm a significant association, citing substantial heterogeneity among the studies analyzed [10].

These conflicting findings highlight the need for further research to better understand the potential role of SES in geographic and demographic variations in DGBI prevalence [9]. Furthermore, clarifying these associations may aid in earlier recognition, targeted management, and reduced healthcare utilization [2]. Therefore, this study aimed to investigate regional differences in DGBI prevalence in the Netherlands and to examine how socio‐economic factors may contribute to these potential variations.

## 2 Materials and Methods

This study was part of the Rome Foundation Global Epidemiology Study (RFGES), a large‐scale multinational study examining the prevalence and associated factors of 22 DGBI across 33 countries spanning six continents. Data collection methods varied by country: online surveys were conducted in 26 countries, face‐to‐face interviews in nine, with both methods used in China and Turkey. A detailed description of the RFGES methodology is available elsewhere [1].

### 2.1 Participants

The Netherlands was one of the 26 countries participating in the RFGES, which employed an anonymous online survey. Participants were recruited through a professional survey company (Qualtrics LLC, Provo, Utah, USA) and were compensated with redeemable points for gifts [1]. A minimum of 2000 respondents were selected based on prespecified demographic criteria, including an equal gender distribution (50% male, 50% female) and an age distribution of 40% aged 18–39 years, 40% aged 40–64 years, and 20% aged 65 and older.

### 2.2 Internet Survey

The internet survey, administered in Dutch, contained up to 160 questions, with the exact number varying depending on participants' responses. The Dutch version of the RFGES questionnaire was translated and validated as part of the Rome Foundation's standardized translation and linguistic validation procedures by TransPerfect Inc. (New York, NY), a professional translation company [13]. It included the complete Rome IV Adult Diagnostic Questionnaire, sociodemographic items, and factors potentially associated with DGBI prevalence. To ensure data quality, the survey incorporated several quality‐assurance measures, including attention‐check questions, completion time thresholds, and repeated questions to detect inconsistent responses. In addition, all questions were mandatory and automatic skip patterns were used, further minimizing the risk of missing or inaccurate data [1, 14]. The study was reviewed by Institutional Review Boards (IRBs) and ethics committees in the various countries surveyed, and deemed exempt from ethics oversight due to the anonymous data collection. However, electronic informed consent was obtained from all participants.

### 2.3 Study Questionnaires

The Rome IV Adult Diagnostic Questionnaire comprised 86 items assessing gastrointestinal symptoms to identify participants with a DGBI. Diagnosed DGBI were classified into six major Rome classification categories: esophageal disorders, gastroduodenal disorders, bowel disorders, anorectal disorders, biliary disorders, and centrally mediated abdominal pain syndrome. Participants with a self‐reported history of organic diseases that could potentially explain their symptoms (e.g., celiac disease, inflammatory bowel disease) were excluded from receiving a DGBI diagnosis. Sociodemographic questions covered age, sex, ethnicity, place of residence, community size, years of education, and relationship status. Additional items covered medical history, healthcare utilization, living conditions, employment status, psychosocial factors, dietary habits, and quality of life, assessed using validated instruments.

### 2.4 Geographic and Socio‐Cultural Regional Division

For the purposes of this study, the Netherlands was divided into three regions based on geographical location and socio‐cultural characteristics. Region 1 (South: provinces Zeeland, North Brabant, Limburg) is characterized by a relatively homogeneous population, limited migration, and a historically Catholic background [15, 16]. Region 2 (West: provinces Utrecht, North Holland, South Holland) is the most culturally diverse and economically developed region in the country [16, 17]. Region 3 (North‐East: provinces Overijssel, Gelderland, Flevoland, Drenthe, Friesland, Groningen) is predominantly rural, with a traditionally strong agricultural foundation [16, 18]. Region 2 was hypothesized to have the highest DGBI prevalence due to its higher urbanization, greater cultural diversity, and lower socio‐cultural cohesion, which may contribute to elevated stress levels [17, 19].

### 2.5 Socio‐Economic Status

Socio‐economic status was estimated using proxy indicators, including years of education, community size (“Up to 50,000” and “50,000 and above”), current and childhood living conditions (“Poor,” “Average,” and “Good”), access to healthcare (“No access,” “Limited access,” and “Full access”), and work status (“Employed” and “Unemployed”). Further details on the indicators used for living conditions and healthcare access can be found in Supporting Information S1.1. Relationship status (“Single,” “Married,” “Divorced,” “Widowed,” and “Co‐habiting”) was also recorded as a potential social determinant. A standardized SES index could not be applied due to the absence of key variables such as household income.

### 2.6 Statistical Analyses

To ensure adequate power for detecting regional differences in the prevalence of DGBI, the precision of prevalence estimates was evaluated at the regional level. If the regional prevalence was 50%, an estimated sample size of 500 participants per region would yield a 95% confidence interval with a margin of error of 4.4%, while a sample size of 800 would reduce this margin to 3.5%. If the true prevalence deviated from 50%, the margin of error would be even smaller. Based on these estimates, the sample size was considered sufficient to allow for meaningful comparisons across regions.

Data were analyzed using R Statistics (Version 4.4.1) [20]. Continuous and discrete count variables were presented as medians with interquartile ranges (IQRs) due to their asymmetrical distributions, while categorical variables were summarized as frequencies with percentages. Differences in baseline characteristics (i.e., age, sex, BMI) and SES factors between the three regions were assessed using the Kruskal–Wallis test for continuous variables and Fisher's exact test for categorical variables. Statistical significance was defined as *p* < 0.05.

For the primary analysis, prevalence with 95% confidence intervals (CIs) were calculated for any DGBI, as well as for each Rome classification group and subtype within each region, using the Wilson method [21]. Regions were compared using Fisher's exact test, with the presence or absence of the DGBI as the dependent variable and region as independent variable. To correct for multiple testing, the Holm‐Bonferroni correction was applied.

To examine the role of socio‐economic factors in DGBI prevalence, a fixed‐effects logistic regression model was applied. This approach was chosen to account for the nested data structure (individuals within regions), given the small number of clusters (three regions) [22]. A hierarchical modeling approach was applied, with the presence of any DGBI as the dependent variable, SES factors as predictors, and region included as fixed effect (i.e., with dummy coding). Age, sex, and body mass index (BMI) were considered as potential confounders. Model assumptions were evaluated and addressed when violated. The best model was selected based on the Akaike information criterion (AIC) and Bayesian information criterion (BIC). For nested models, likelihood ratio tests (LRT) were conducted to assess whether inclusion of additional predictors significantly improved model fit. Predictive performance of the best model according to AIC, BIC, and LRT, was further evaluated using ROC/AUROC analysis. Results are reported as odds ratios (ORs) with corresponding 95% CIs and (Wald) *p*‐values. For exploratory purposes, the influence of SES factors on DGBI group prevalence was also examined using fully adjusted models, with DGBI groups ordered by decreasing prevalence.

Missing data were assessed by variable and possible predictors of missingness were examined for each variable with missing data using logistic regression (see Supporting Information S1.1). Outcomes (i.e., any DGBI, DGBI groups and subtypes), independent variables (i.e., region, SES factors, age, sex, and BMI), and predictors related to missingness were included in the multiple imputation model. Imputation was performed using the mice package, applying predictive mean matching with 15 imputations (*m* = 15) and 30 iterations to ensure model convergence [23]. Both complete case analysis and multiple imputation were conducted using the same statistical approach and yielded similar results. The results presented are based on the multiple imputation analysis. Findings from the complete case analysis and additional details on statistical choices are provided in Supporting Information S1.1 and S2.2.

## 3 Results

### 3.1 Participants

A total of 2008 participants completed the internet survey, equally split between males and females (1004 each) as per the prespecified demographic criteria. The median age was 48 years (IQR: 31), and the median BMI was 25.25 kg/m^2^ (IQR: 5.98). Most participants (95.37%) identified as Dutch. Among the various other reported backgrounds, the most common were Surinamese (0.55%), German (0.50%), Belgian (0.45%), and Indonesian (0.45%). Regional origin was available for 2007 participants: 523 participants (26.06%) resided in Region 1, 848 (42.25%) in Region 2, and 636 (31.69%) in Region 3. No significant demographic differences were observed between regions. Detailed characteristics by region are presented in Table 1.

**TABLE 1:** Baseline characteristics across regions.

Characteristic	Region 1 (*n* = 523)	Region 2 (*n* = 848)	Region 3 (*n* = 636)	*p*
Sex				0.105
Male	276 (52.77%)	401 (47.29%)	326 (51.26%)	
Female	247 (47.23%)	447 (52.71%)	310 (48.74%)	
Age, years	50.00 (30.00)	48.00 (31.00)	47.00 (31.25)	0.067
BMI, kg/m^2^	25.35 (6.04)	25.20 (6.02)	24.98 (5.91)	0.588

*Note:* Baseline characteristics are presented based on the complete case analysis. Values are reported as proportions (percentages) and medians (IQR) unless stated otherwise. Fisher's exact test was used to compare gender distribution across regions, and the Kruskal–Wallis test was used to compare age and BMI across regions.

Abbreviation: BMI, body mass index.

### 3.2 Regional Prevalence of Any DGBI Within the Netherlands

**FIGURE 1:**
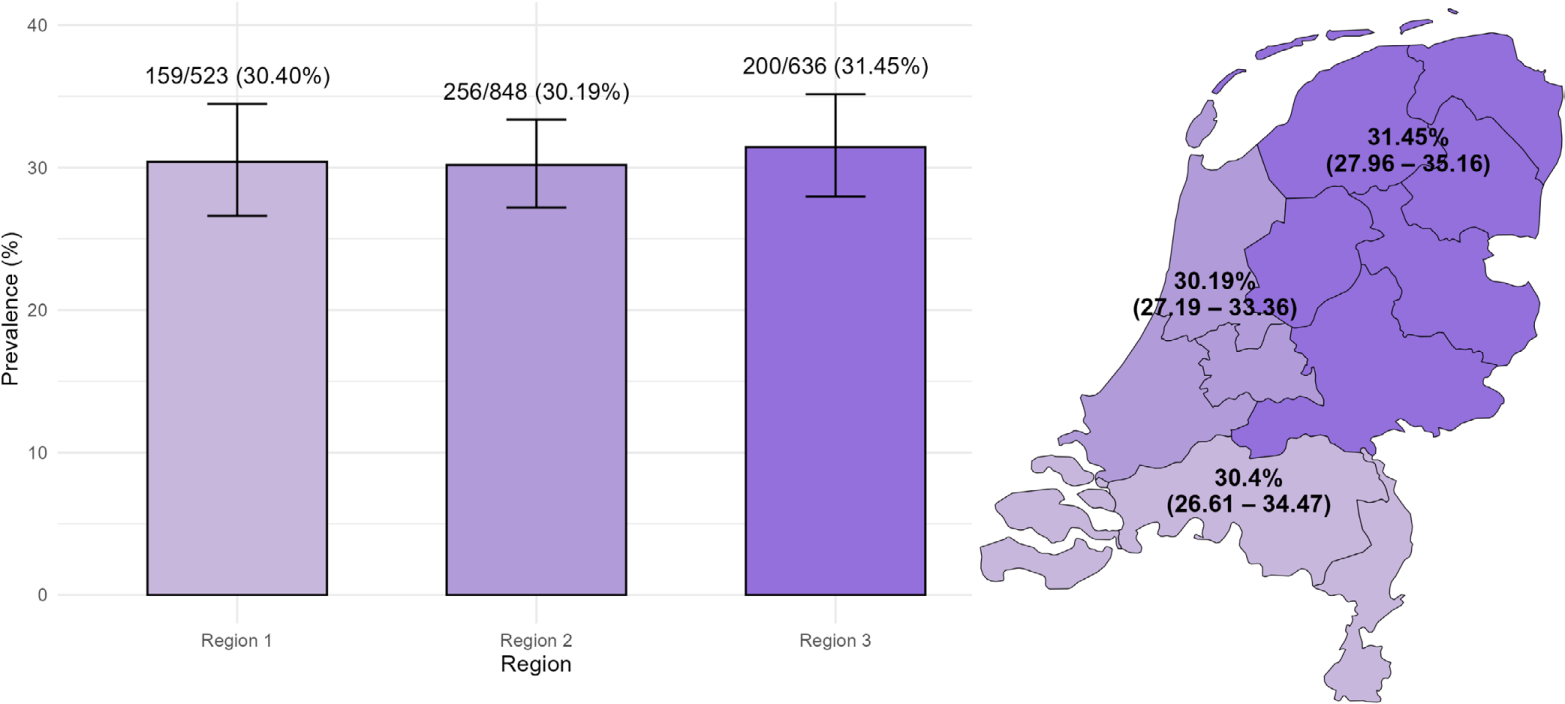
DGBI prevalence by region in the Netherlands. The prevalence of any DGBI per region in the Netherlands. Regions were defined as: Region 1 (South), Region 2 (West), and Region 3 (North‐East).

The overall prevalence of any DGBI in the Netherlands was estimated at 30.63% (95% CI: 28.65–32.68), with bowel disorders (26.20%, 95% CI: 24.32–28.16) being the most prevalent, followed by gastroduodenal (6.08%, 95% CI: 5.11–7.21), esophageal (4.68%, 95% CI: 3.84–5.69), anorectal (4.43%, 95% CI: 3.62–5.42), and biliary (0.05%, 95% CI: 0.01–0.28) disorders. Regional prevalence proportions were as follows: 159 individuals in Region 1 (30.40%, 95% CI: 26.61–34.47), 256 in Region 2 (30.19%, 95% CI: 27.19–33.36), and 200 in Region 3 (31.45%, 95% CI: 27.96–35.16). No statistically significant differences in prevalence were observed across the regions (*p* = 1.000); see Figure 1.

### 3.3 Regional Prevalence of DGBI Groups and Subtypes in the Netherlands

**FIGURE 2:**
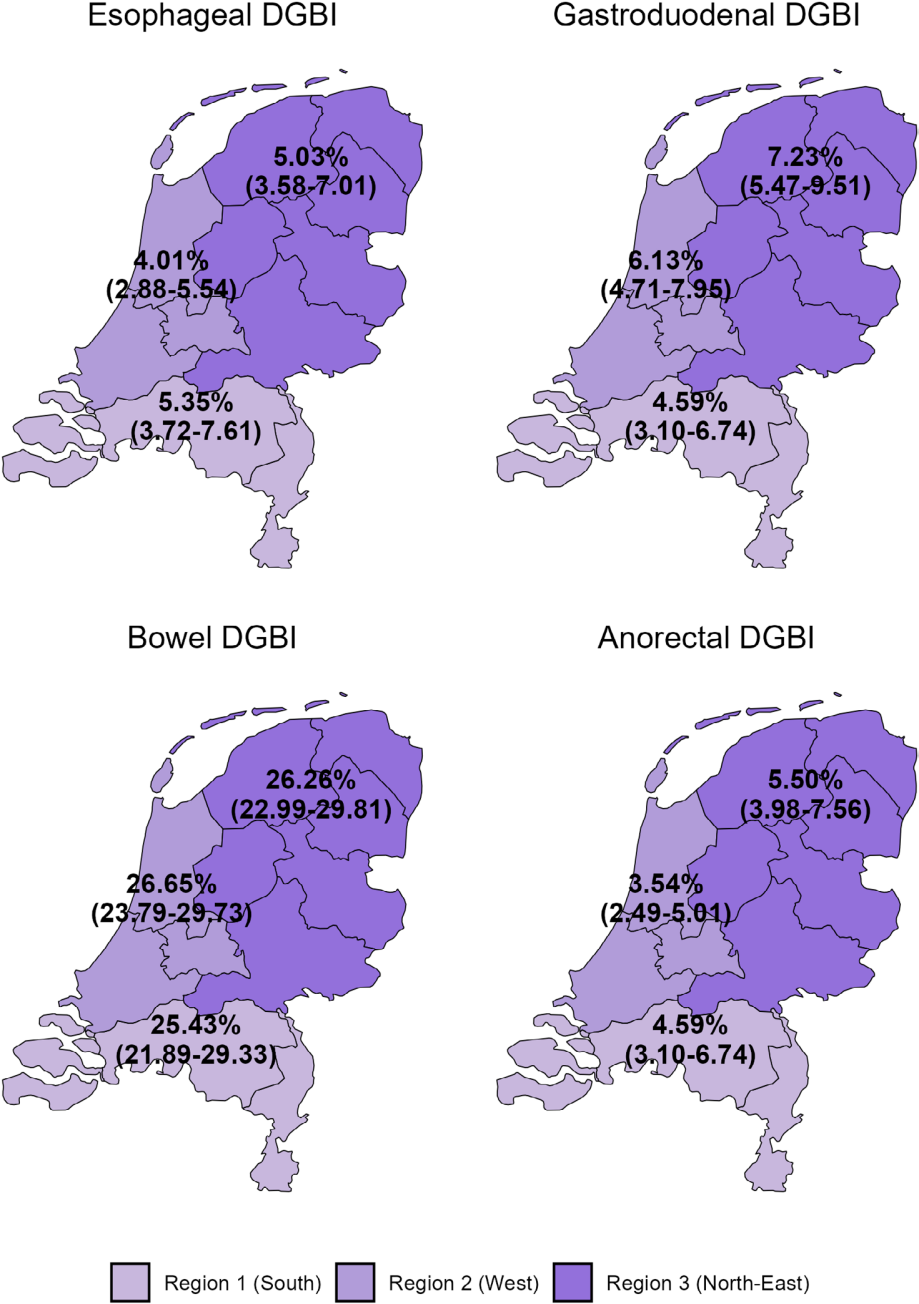
DGBI group prevalence by region in the Netherlands. The prevalence estimates of the DGBI Rome classification groups per region in the Netherlands. Prevalence of Biliary DGBI and Central GI Pain DGBI was 0.00% in all regions (no cases observed). Regions were defined as: Region 1 (South), Region 2 (West), and Region 3 (North‐East).

Regional prevalence was estimated for the six DGBI Rome subclassification groups. No statistically significant differences were observed between regions for esophageal (*p* = 1.000), gastroduodenal (*p* = 1.000), bowel (*p* = 1.000), anorectal (*p* = 1.000), or biliary DGBI (*p* = 1.000). No participants were identified with centrally mediated abdominal pain syndrome. Similarly, no significant regional differences were found for any individual DGBI subtype. Detailed prevalence estimates by region are presented in Table 2 and Figure 2.

**TABLE 2:** Regional prevalence of DGBI groups and subtypes in the Netherlands.

DGBI	Region 1 (*n* = 523)	95% CI	Region 2 (*n* = 848)	95% CI	Region (*n* = 636)	95% CI	*p*
Esophageal disorders							
Overall esophageal disorders	28 (5.35%)	3.72–7.61	34 (4.01%)	2.88–5.54	32 (5.03%)	3.58–7.01	1.000
Functional heartburn	2 (0.38%)	0.10–1.38	2 (0.24%)	0.06–0.86	4 (0.63%)	0.24–1.61	1.000
Functional chest pain	11 (2.10%)	1.18–3.73	10 (1.18%)	0.64–2.16	11 (1.73%)	0.97–3.07	1.000
Reflux hypersensitivity	2 (0.38%)	0.10–1.38	2 (0.24%)	0.06–0.86	3 (0.47%)	0.16–1.38	1.000
Globus	4 (0.76%)	0.30–1.95	8 (0.94%)	0.48–1.85	0 (0.00%)	0.00–0.60	0.599
Dysphagia	12 (2.29%)	1.32–3.97	15 (1.77%)	1.07–2.90	17 (2.67%)	1.68–4.24	1.000
Gastroduodenal disorders							
Overall gastroduodenal disorders	24 (4.59%)	3.10–6.74	52 (6.13%)	4.71–7.95	46 (7.23%)	5.47–9.51	1.000
Functional dyspepsia	13 (2.49%)	1.46–4.21	38 (4.48%)	3.28–6.09	31 (4.87%)	3.45–6.84	1.000
PDS	9 (1.72%)	0.91–3.24	32 (3.77%)	2.69–5.28	25 (3.93%)	2.68–5.74	1.000
EPS	6 (1.15%)	0.53–2.48	9 (1.06%)	0.56–2.00	9 (1.42%)	0.75–2.67	1.000
Belching	0 (0.00%)	0.00–0.73	2 (0.24%)	0.06–0.86	1 (0.16%)	0.03–0.89	1.000
Rumination	10 (1.91%)	1.04–3.48	12 (1.42%)	0.81–2.46	12 (1.89%)	1.08–3.27	1.000
CNVS	5 (0.96%)	0.41–2.22	4 (0.47%)	0.18–1.21	5 (0.79%)	0.34–1.83	1.000
Cyclic vomiting	0 (0.00%)	0.00–0.73	4 (0.47%)	0.18–1.21	5 (0.79%)	0.34–1.83	1.000
Cannabinoid hyperemesis syndrome	0 (0.00%)	0.00–0.73	0 (0.00%)	0.00–0.45	0 (0.00%)	0.00–0.60	NA
Bowel disorders							
Overall bowel disorders	133 (25.43%)	21.89–29.33	226 (26.65%)	23.79–29.73	167 (26.26%)	22.99–29.81	1.000
IBS	22 (4.21%)	2.79–6.29	34 (4.01%)	2.88–5.55	20 (3.14%)	2.04–4.81	1.000
Functional constipation	53 (10.13%)	7.83–13.02	75 (8.84%)	7.11–10.95	57 (8.96%)	6.98–11.44	1.000
Opioid induced constipation	1 (0.19%)	0.03–1.08	8 (0.94%)	0.48–1.85	7 (1.10%)	0.53–2.25	1.000
Functional diarrhea	17 (3.25%)	2.04–5.14	18 (2.12%)	1.35–3.33	30 (4.72%)	3.32–6.65	0.521
Functional bloating	5 (0.96%)	0.41–2.22	14 (1.65%)	0.99–2.75	8 (1.26%)	0.64–2.46	1.000
Functional bowel disorder, unspecified	36 (6.88%)	5.01–9.38	80 (9.43%)	7.65–11.59	46 (7.23%)	5.47–9.51	1.000
Anorectal disorders							
Overall anorectal disorders	24 (4.59%)	3.10–6.74	30 (3.54%)	2.49–5.01	35 (5.50%)	3.98–7.56	1.000
Fecal incontinence	11 (2.10%)	1.18–3.73	12 (1.42%)	0.81–2.46	10 (1.57%)	0.86–2.87	1.000
Levator ani	4 (0.76%)	0.30–1.95	8 (0.94%)	0.48–1.85	6 (0.94%)	0.43–2.04	1.000
Proctalgia fugax	11 (2.10%)	1.18–3.73	10 (1.18%)	0.64–2.16	22 (3.46%)	2.30–5.18	0.322
Biliary disorders							
Functional biliary pain	0 (0.00%)	0.00–0.73	0 (0.00%)	0.00–0.45	1 (0.16%)	0.03–0.89	1.000
Central GI pain disorders							
CAPS	0 (0.00%)	0.00–0.73	0 (0.00%)	0.00–0.45	0 (0.00%)	0.00–0.60	NA

*Note:* Fisher's exact test was used to compare DGBI group and subtype prevalence across regions; 95% confidence intervals were calculated using the Wilson method. All *p*‐values were corrected for multiple testing using the Holm–Bonferroni correction method.

Abbreviations: CAPS, chronic abdominal pain syndrome; CNVS, chronic nausea and vomiting syndrome; DGBI, disorders of gut‐brain interaction; EPS, epigastric pain syndrome; IBS, irritable bowel syndrome; NA, not applicable; PDS, postprandial distress syndrome.

### 3.4 Regional Differences in Socio‐Economic Status

Significant regional differences were found in participants' community size (*p* < 0.0001), current living conditions (*p* < 0.0001), childhood living conditions (*p* = 0.017), and relationship status (*p* = 0.011) (Table 3). Post hoc analyses indicated differences in community size between Regions 1 and 2, and Regions 2 and 3; current living conditions differed across all three regions; childhood living conditions differed between Regions 1 and 2, and Regions 2 and 3; and relationship status differed between Regions 1 and 3, and Regions 2 and 3. No regional differences were found for access to healthcare (*p* = 0.200) or work status (*p* = 0.939).

**TABLE 3:** Regional differences in socio‐economic status.

SES factor	Region 1 (*n* = 523)	Region 2 (*n* = 848)	Region 3 (*n* = 636)	*p*
Education, years	14.00 (5.00)	15.00 (6.00)	15.00 (6.00)	0.051
Community size				< 0.0001*
Up to 50,000 inhabitants	374 (71.51%)	415 (48.94%)	436 (68.55%)	
50,000 and above inhabitants	149 (28.49%)	433 (51.06%)	200 (31.45%)	
Current living conditions				< 0.0001**
Poor	34 (6.50%)	30 (3.54%)	19 (2.99%)	
Average	181 (34.61%)	385 (45.40%)	236 (37.11%)	
Good	308 (58.89%)	433 (51.06%)	381 (59.91%)	
Childhood living conditions				0.017***
Poor	53 (10.13%)	52 (6.13%)	59 (9.28%)	
Average	294 (56.21%)	502 (59.20%)	388 (61.01%)	
Good	176 (33.65%)	294 (34.67%)	189 (29.72%)	
Access to health care				0.200
No access	1 (0.19%)	7 (0.83%)	4 (0.63%)	
Limited access	31 (5.93%)	31 (3.66%)	30 (4.72%)	
Full access	491 (93.88%)	810 (95.52%)	602 (94.65%)	
Work status				0.939
Unemployed	243 (46.46%)	388 (45.75%)	289 (45.44%)	
Employed	280 (53.54%)	460 (54.25%)	347 (54.56%)	
Relationship status				0.011****
Single	146 (27.92%)	280 (33.02%)	197 (30.97%)	
Married	242 (46.27%)	363 (42.81%)	310 (48.74%)	
Divorced	36 (6.88%)	54 (6.37%)	17 (2.67%)	
Widowed	15 (2.87%)	22 (2.59%)	13 (2.04%)	
Co‐habiting (not married but living with an adult partner)	84 (16.06%)	129 (15.21%)	99 (15.57%)	

*Note:* SES factors are presented based on the complete case analysis. Values are reported as proportions (percentages) and medians (IQR) unless stated otherwise. The Kruskal–Wallis test was used to compare education across regions, and Fisher's exact test was used to compare categorical SES factors across regions.

Abbreviation: SES, socio‐economic status.

* Regions 1 versus 2: *p* < 0.0001; Regions 1 versus 3: *p* = 0.303; Regions 2 versus 3: *p* < 0.0001.

** Regions 1 versus 2: *p* < 0.0001; Regions 1 versus 3: *p* = 0.016; Regions 2 versus 3: *p* = 0.003.

*** Regions 1 versus 2: *p* = 0.027; Regions 1 versus 3: *p* = 0.255; Regions 2 versus 3: *p* = 0.021.

**** Regions 1 versus 2: *p* = 0.401; Regions 1 versus 3: *p* = 0.010; Regions 2 versus 3: *p* = 0.006.

### 3.5 Socio‐Economic Influence on Any DGBI Prevalence

A hierarchical modeling approach was applied to assess the association between SES and DGBI prevalence (Supporting Information S2.1). Model fit improved sequentially across nested models, with the fully adjusted model demonstrating the best fit based on AIC and BIC. Likelihood ratio tests confirmed that the addition of SES and demographic predictors significantly improved model fit (LRT = 21.05, df = 5, *p* < 0.0001). Predictive performance, assessed using ROC analysis, showed a median AUROC of 0.68, indicating fair discriminative ability. These results suggests that SES and demographic factors contributed meaningfully to explaining variation in DGBI prevalence. Model assumptions were checked, and the linearity assumption for BMI was not met. Hence, BMI was categorized into four groups based on WHO definitions [24]. No other violations were detected.

**FIGURE 3:**
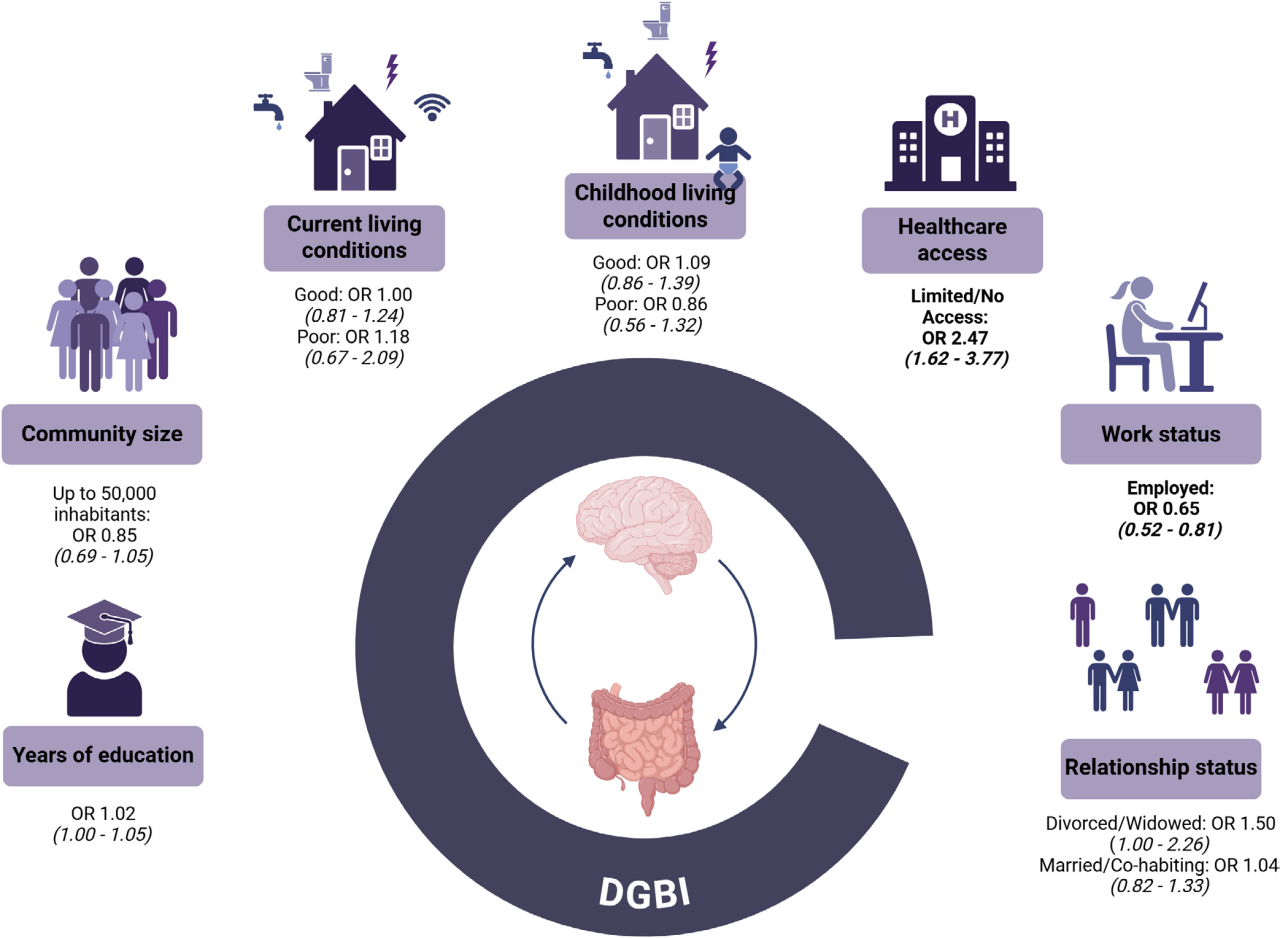
Socio‐economic influence on any DGBI prevalence. The association between proxy indicators of socio‐economic status and the prevalence of any DGBI. Variables include years of education, community size (up to 50,000 inhabitants, *reference: 50,000 and above inhabitants*), current living conditions (good or poor, *reference: Average*), childhood living conditions (good or poor, *reference: Average*), healthcare access (limited or none, *reference: Full access*), work status (employed, *reference: Unemployed*), and relationship status (divorced/widowed or married/cohabiting, *reference: Single*). Statistically significant and clinically relevant odds ratios are highlighted in bold. This figure was created with BioRender.com.

In the final model, adjusted for age, sex, and BMI, limited or no access to healthcare was strongly associated with increased odds of having any DGBI (OR = 2.47, 95% CI [1.62–3.77], *p* < 0.0001). Conversely, being employed was associated with decreased odds (OR = 0.65, 95% CI [0.52–0.81], *p* < 0.001). Other SES factors were not significantly associated with DGBI prevalence. Younger age (OR = 0.98, 95% CI [0.98–0.99], *p* < 0.0001), female sex (OR = 2.10, 95% CI [1.70–2.59], *p* < 0.0001), underweight (OR 2.53, 95% CI [1.36–4.73]), and obesity (OR 1.59, 95% CI [1.19–2.13]) were all significantly associated with higher odds of any DGBI (see Table 4, Figure 3).

**TABLE 4:** Socio‐economic influence on DGBI prevalence.

	OR	95% CI	*p*
Region 2	0.89	0.69–1.14	0.352
Region 3	1.02	0.78–1.33	0.872
Education (years)	1.02	1.00–1.05	0.068
Community size			
Up to 50,000 inhabitants	0.85	0.69–1.05	0.123
Current living conditions			
Good	1.00	0.81–1.24	0.975
Poor	1.18	0.67–2.09	0.563
Childhood living conditions			
Good	1.09	0.86–1.39	0.461
Poor	0.86	0.56–1.32	0.495
Healthcare access			
Limited/no access	2.47	1.62–3.77	**< 0.0001**
Work status			
Employed	0.65	0.52–0.81	**< 0.001**
Relationship status			
Divorced/widowed	1.50	1.00–2.26	0.051
Married/co‐habiting	1.04	0.82–1.33	0.735
Age	0.98	0.98–0.99	**< 0.0001**
Sex			
Female	2.10	1.70–2.59	**< 0.0001**
BMI			
Underweight	2.53	1.36–4.73	**0.004**
Overweight	0.95	0.74–1.22	0.692
Obese	1.59	1.19–2.13	**0.002**

*Note:* This table presents the fully adjusted model examining the influence of socio‐economic factors on DGBI prevalence, including any DGBI, region, socio‐economic factors, and age, sex, and BMI as potential confounders. These findings are based on multiple imputation analysis. Model fit: AIC = 2337, BIC = 2438. Median AUROC = 0.68. References categories: Region (Region 1), Community size (50,000 and above inhabitants), Living conditions (average), Living conditions childhood (average), Healthcare access (full access), Work status (unemployed), Relationship status (single), Sex (male), BMI (normal weight). BMI was categorized based on the WHO categories. The numbers highlighted in bold indicate the statistical significance of these values.

Abbreviations: 95% CI, 95% confidence interval; BMI, body mass index; OR, odds ratio.

### 3.6 Socio‐Economic Influence on DGBI Group Prevalence

The influence of SES on individual DGBI groups was explored using fully adjusted models only, ordered by decreasing prevalence (see Supporting Information S2.1.2). No violations of model assumptions were detected. For bowel DGBI, clinically and significantly relevant predictors included limited or no access to healthcare (OR 2.10, 95% CI [1.37–3.21], *p* < 0.001), employment (OR 0.68, 95% CI [0.54–0.86], *p* = 0.001), female sex (OR 2.19, 95% CI [1.75–2.72], *p* < 0.0001), and BMI (Underweight: OR 2.03, 95% CI [1.10–3.73], *p* = 0.023; Obese: OR 1.56, 95% CI [1.16–2.11], *p* = 0.004). For gastroduodenal DGBI, associations were found for limited or no access to healthcare (OR 1.97, 95% CI [1.04–3.72], *p* = 0.037), being employed (OR 0.62, 95% CI [0.41–0.93], *p* = 0.022), and BMI (Underweight: OR 5.09, 95% CI [2.48–10.43], *p* < 0.0001). For esophageal DGBI being employed was associated with lower odds (OR 0.56, 95% CI [0.35–0.89], *p* = 0.015). For anorectal DGBI, predictors included smaller community size (OR 0.56, 95% CI [0.36–0.88], *p* = 0.012), limited or no access to healthcare (OR 3.31, 95% CI [1.70–6.44], *p* < 0.001), and BMI (Underweight: OR 2.97, 95% CI [1.10–7.98], *p* = 0.031; Obese: OR 2.08, 95% CI [1.15–3.78], *p* = 0.016).

## 4 Discussion

In this nationwide cohort, 30.63% of adults in the Netherlands met the criteria for at least one DGBI, with consistent regional distribution across the prevalence of any DGBI, Rome classification groups, and subtypes. SES differed by regions in terms of community size, living conditions (current and childhood), and relationship status. Limited or no access to healthcare and unemployment were significantly associated with higher odds of having any DGBI. Demographic factors, including age, sex, and BMI, were also independently associated with DGBI. Exploratory analyses suggests that specific SES variables may be differentially associated across Rome classification groups.

Historically, the prevalence of DGBI has been poorly reported, as prior epidemiological studies have largely focused on IBS and functional dyspepsia, with limited attention to the full spectrum of DGBI [10, 25, 26]. The Rome Foundation Global Epidemiology study has since provided reliable, world‐wide prevalence estimates [1]. Globally, 40.3% of individuals were affected by at least one DGBI, whereas the cohort from the Netherlands showed a notably lower DGBI prevalence of 30.63%. One possible explanation for this discrepancy may be the limited ethnic diversity in the sampled population. In this cohort, 95.37% of participants identified as Dutch, which does not accurately reflect the demographic composition of the Netherlands, particularly in the large urban areas where approximately half of the population has a non‐Western migration background [19, 27]. Consequently, this sample may not fully capture the heterogeneity of the population in the Netherlands.

Prevalence rates may be higher among underrepresented groups. For instance, individuals with a Turkish background, who constitute a substantial share of the urban population in the Netherlands, accounted for only 0.10% of the sample. Given that Sperber et al. [1] reported a DGBI prevalence of 39.7% in Turkey, closer to global estimates, it is possible that the underrepresentation of certain ethnic groups with a potentially higher prevalence may have contributed to an underestimation of the true DGBI burden in the Netherlands. However, cross‐country comparisons should be interpreted with caution. Furthermore, psychosocial stressors associated with migration may also contribute to higher DGBI prevalence rates in underrepresented groups [19, 28].

Nevertheless, even if the true prevalence is underestimated, the reported prevalence in the Netherlands remains considerable, particularly given the substantial burden that DGBI impose on patients' quality of life, emotional well‐being, and healthcare utilization [4]. Specifically, in the Netherlands, bowel disorders were the most prevalent classification group (26.2%), followed by gastroduodenal (6.08%), esophageal (4.68%), anorectal (4.43%), and biliary (0.05%) disorders, patterns that are consistent with global trends [1].

Regional prevalences in the Netherlands ranged from 30.19% (Region 2) to 31.45% (Region 3). Despite differences in geographical location and socio‐cultural characteristics, no significant differences were observed between regions for any DGBI, DGBI groups, and subtypes. Region 2, which includes Utrecht, North Holland, and South Holland, was hypothesized to have the highest DGBI prevalence, given its higher levels of urbanization, greater cultural diversity, and lower socio‐cultural cohesion [17, 19]. These factors were expected to contribute to increased psychosocial stress, which may alter gut‐brain axis signaling and thereby increase the risk of DGBI [4, 29]. However, this hypothesis was not supported by the findings. One possible explanation for the lack of regional differences may be the limited ethnic diversity of the sample, as discussed above, which could have attenuated the potential influence of cultural variation. Supporting the role of cultural context, Broeders et al. [30] reported differences in DGBI prevalence and quality‐of‐life impact between French‐speaking and Dutch‐speaking populations in Belgium, indicating that cultural variation can meaningfully influence DGBI burden. While DGBI prevalence is generally thought to be shaped by cultural context and country‐specific factors, the present findings suggest that socio‐cultural differences may exert only a limited influence at the regional level within the Dutch context [9]. Hence, a uniform, nationwide approach to DGBI care (and healthcare in general) may be more effective than region‐specific strategies and could help ensure consistent management across regions.

Determinants of socioeconomic status may be considered in these strategies. While causality cannot be inferred from our results, SES factors may act as contributing factors to DGBI prevalence and warrant attention in clinical care. Specifically, our findings showed that limited or no access to healthcare and unemployment were independently associated with increased odds of having a DGBI. This may reflect that individuals with inadequate access to healthcare may be at greater risk of not receiving appropriate medical evaluation and guidance, particularly for conditions like DGBI, which rely primarily on symptom‐based diagnosis and patient‐clinician interaction [31, 32]. This could contribute to delayed diagnoses, mismanagement, or worsened disease outcomes [32, 33].

Notably, despite healthcare access and employment status being considered potential contributors to DGBI prevalence, their uniform distribution across regions may help explain the paradoxical lack of observed regional differences in DGBI prevalence. These findings suggest that when key risk factors are evenly distributed or counterbalanced by other regional factors, for example in case of a region with poorer healthcare access but higher employment, the net effect on DGBI prevalence may appear similar across regions. Furthermore, unmeasured confounders at the individual level, such as mental health or dietary habits, may also contribute [1, 4, 30]. Further research should explore factors that could act as protective or modifying influences on regional DGBI prevalence.

The finding regarding healthcare access is particularly noteworthy in the Dutch context, where health insurance coverage is nearly universal, suggesting that insurance alone may not eliminate all barriers to adequate care. While insurance may be widespread, SES can still affect access to timely and adequate care by influencing patient's healthcare‐seeking behavior, health beliefs, and the quality of communication with physicians [34, 35, 36, 37]. For example, an interview study by Bernheim et al. [34] showed that patients' SES affected clinical decision‐making, with lower SES linked to increased strain and suboptimal management. Enhancing communication strategies, through increased physician awareness of SES‐related differences and by empowering patients to express concerns and preferences, may represent an important first step toward improving DGBI care and reducing SES‐related disparities [37].

Unemployment has long been recognized as a key contributor to poor health outcomes and health inequalities, even when adjusted for social class, poverty, age, and pre‐existing conditions [38, 39, 40]. This association is likely driven by the psychological and social consequences of job loss, including financial strain, increased stress, and a higher prevalence of mental health issues [38]. Psychological comorbidities such as anxiety and depression are highly prevalent in individuals with DGBI, and may partly mediate the relationship between socio‐economic disadvantage and DGBI risk [9]. Indeed, low SES, particularly unemployment, has been identified as a factor associated with elevated anxiety in this population [4, 41, 42]. Nunes et al. [42] further showed that low income and limited education were associated with higher scores on the General Anxiety Disorder‐7 (GAD‐7) questionnaire. However, our results do not support an association between years of education and DGBI. Addressing these issues through policies that promote financial security, improved access to proactive healthcare, and providing opportunities for retraining could help mitigate the adverse health effects of unemployment and reduce associated DGBI risk [39].

Other SES factors, including community size, current and childhood living conditions, years of education, and relationship status, were not associated with higher odds of having any DGBI. Nevertheless, previous research has suggested that early‐life socioeconomic disadvantages may have ongoing biological effects and influence adult physiology [9, 43]. Evidence from genome‐wide transcriptional analyses of blood samples demonstrated that lower childhood SES was associated with persistent upregulation of inflammatory gene expression in adulthood, independent of adult socioeconomic conditions [43]. Although our findings did not support this association, they do not preclude the possibility that early‐life SES could contribute to DGBI risk in later life. Additionally, our exploratory analyses suggest that specific SES variables may be differentially associated across the DGBI Rome classification groups. These findings warrant further investigation, particularly given the relatively low prevalence rates across subgroups in the cohort from the Netherlands.

Demographic factors, including age, sex, and BMI, were included as potential confounders in our model and were all independently associated with DGBI. This aligns with previous research, which has shown that female sex, younger age, and both underweight and obesity are associated with increased DGBI prevalence [1, 4, 44, 45]. However, DGBI rates are also high in people of older age and necessitate clinical awareness [44]. Notably, such contributing factors may not operate in isolation but can interact in complex ways with SES [9]. For example, a study by Husain et al. [46] found higher IBS risk among men with high income but fewer years of education, based on sex‐stratified analysis. This underscores the importance of examining the combined effects of socioeconomic and demographic variables on DGBI risk.

The overall effect of SES on DGBI prevalence was not investigated in this study, as a validated composite SES index could not be applied due to the absence of key components (e.g., household income) in the survey. Instead, individual SES variables were analyzed separately. This limitation, common in epidemiological research, reflects the broader challenge of accurately capturing economic data at the individual level, which are often prone to inaccuracy and bias [9, 10]. Consequently, SES is frequently inferred through indirect indicators, such as educational level or housing type, which may not fully reflect an individual's overall economic status [10, 46, 47]. To date, only one meta‐analysis including four studies has examined the association between overall SES and IBS prevalence, and found no significant differences across SES groups [10]. However, this evidence is limited in scope, both in terms of the number of studies and its focus on IBS alone. Future research should prioritize investigating the role of composite SES measures in relation to the full spectrum of DGBI.

Beyond the constraints in assessing overall SES, other methodological limitations should also be acknowledged. First, data were derived from a large‐scale epidemiological study utilizing an online, non‐clinical questionnaire. As such, symptoms were self‐reported and not clinically confirmed, introducing the possibility that some participants had an organic cause of gastrointestinal symptoms rather than DGBI. However, this risk was partially mitigated by incorporating a checklist of organic diseases and excluding such cases from DGBI prevalence estimates, while keeping them in the denominator. Furthermore, recruitment of participants through an internet survey panel may have introduced selection bias. In addition, as the survey was administered only in Dutch, it may have limited the inclusion of participants with limited language proficiency. Furthermore, some data were missing; however, the extent was limited (10.51%), and results were consistent between complete case analysis and multiple imputation, supporting the robustness of the findings. A key limitation inherent to multiple imputation is its reliance on the assumption that data are missing at random (MAR), an assumption that cannot be empirically verified [48]. To enhance the plausibility of this assumption, we included all predictors associated with missingness in the imputation model (for details, see Supporting Information S1.1).

To conclude, this study provides valuable regional prevalence estimates of DGBI in the Netherlands. The absence of regional variation supports the implementation of nationwide strategies for their recognition, management, and prevention of DGBI. Socio‐economic indicators, particularly healthcare access and employment status emerged as putative contributing factors and should be considered in DGBI care. Further research employing validated SES indices is warranted to further elucidate the overall contribution of SES to the burden of DGBI.

## Author Contributions

Study concept and implementation of original study: A.D.S., O.S.P., M.A.B, and S.I.B. Study concept and design of Dutch paper: F.V., D.K, and F.I. Data processing, interpretation and statistical analysis: F.V. and F.I. Manuscript writing: F.V. Constructive review of manuscript: F.I., M.A.B., A.D.S., O.S.P., S.I.B., and D.K. Supervisor: D.K. All authors approved the final manuscript.

## Conflicts of Interest

The authors declare no conflicts of interest.

## Supporting information


**Data S1:** nmo70181‐sup‐0001‐Supinfo.docx.

## Data Availability

The data that supports the findings of this study is available in the Supporting Information of this article.
