# Supplementary material for: Socio‐Economic Determinants and Regional Prevalence of Disorders of Gut‐Brain Interaction in the Netherlands: Results From the Rome Foundation Global Epidemiology Study
Source: Neurogastroenterol Motil. 2025 Oct 16;38(1):e70181. doi: 10.1111/nmo.70181 (PMC12814999; doi:10.1111/nmo.70181)
Supplement: Supplementary file 1 — Data S1: nmo70181‐sup‐0001‐Supinfo.docx. [file NMO-38-e70181-s001.docx]

**Supplementary Data**

**1.Methods**

- 1. **Choices made in the statistical analysis**

**Height:**

Range 120-220 cm.

**Weight:**

Range 35-225kg.

**BMI:**

Range 14-55.

For 6 participants with missing values for BMI, the height and weight values are technically valid according to the ranges set by the Rome Foundation. This is because the *individual* values for height and weight fall within acceptable limits. However, the *combination* of these two values results in a BMI that falls outside the plausibility threshold (BMI <14 or >55) and is therefore coded as NA.

**Years of education:**

<31 years.

**Provinces/region:**

1 missing value for 1 participant (2007 participants out of 2008 available for analysis).

**DGBIs**

The diagnostic scoring of DGBIs using the Rome IV Diagnostic Questionnaire did not take into account participant’s self-reported history of organic diseases. Therefore, individuals with diagnoses that could potentially explain their FGID symptoms were excluded from a DGBI diagnoses. Exclusion was made based on guidelines provided by the Global Epidemiology Team:

1. Individuals endorsing celiac disease (s17_7), GI cancer (s17_9), or IBD (s17_8) are to be excluded from all Rome IV FGID diagnoses.
2. Individuals endorsing peptic ulcer disease (s17_11*) are to be excluded from esophageal, gastroduodenal and biliary diagnoses, but can be diagnosed with bowel and anorectal disorders.
3. Individuals endorsing diverticulitis (s17_12) are to be excluded from bowel and anorectal disorders, but not from esophageal, gastroduodenal or biliary diagnoses.
4. Individuals endorsing bowel resection (s18_4) should be excluded from bowel and anorectal disorders, but not from esophageal, gastroduodenal or biliary.

**SES factors**

Community size

Classified into two groups (binary):

- Up to 50,000
- 50,000 and above.

Living conditions

Based on questions:

- **s5:** I live on a farm (s5_1), My home has running water on tap (S5_2), my home has electrical power (S5_3)
- **s6:** amount of people (s6a), bedrooms (s6b), toilets (s6c)
- **s7:** telephone access
- **s8:** internet access.

Classified into 3 categories:

- *Poor:* if any of the basics is missing - running water (s5_2), electricity (s5_3), or toilet (s6c=0), regardless of phone/internet access
- *Average:* has running water (s5_2), electricity (s5_3), exactly 1 toilet (s6c=1), regardless of phone/internet access
- *Good:* has running water (s5_2), electricity (s5_3), 2 or more toilets (s6c >2), and telephone (s7) and internet (s8) access

Living conditions childhood

Based on questions:

- **s9:** I lived on a farm (s9_1), My home had running water on tap (S9_2), my home had electrical power (S9_3)
- **s10:** amount of people (s10a), bedrooms (s10b), toilets (s10c)
- No variables regarding phone or internet access.

Classified into 3 categories:

- *Poor:* if any of the basics is missing - running water (s9_2), electricity (s9_3), or toilet (s10c=0)
- *Average:* has running water (s9_2), electricity (s9_3), exactly 1 toilet (s10c=1)
- *Good:* has running water (s9_2), electricity (s9_3), 2 or more toilets (s10c >2)

Access to healthcare

Based on question:

- **s15: self-reported ability to visit a doctor** - yes, easily (1), yes, but it would be difficult (2), No (0)

The following related variables were considered but ultimately excluded from the “Access to healthcare” composite variable:

- **s13: Type of healthcare typically sought** *- Western style medicine (1), traditional or folk healer (2), Both (3), Neither (4).* This item was excluded because reliance on alternative care may reflect personal preference rather than an actual barrier to accessing conventional healthcare. Including such responses could lead to misclassification of individuals who do have access but opt for non-standard treatments.
- **s14: frequency of doctor visits** *- once a month or more (1), a few times a year (2), once a year (3), less than once a year (4), never (5).* This item was not included as it does not necessarily reflect true access to healthcare. Individuals who do not seek care may still face barriers to healthcare access, while others may have access but choose not to seek care. The exclusion of this variable helps avoid misclassifying individuals who, although not frequent users of healthcare services, are not necessarily experiencing restricted access.
- **s16: Perceived medical expenses** *- not having to pay anything/free access (1), to pay a small fee (2), to pay a substantial part (3), to pay all medical expenses (4).* Although financial burden is an important dimension of access, in the context of the Dutch healthcare system, which ensures widespread coverage and regulates healthcare costs, this item may not reliably differentiate true access. For example, individuals choosing private care despite having full access to public services might be misclassified.

Classified into 3 categories:

- *No access:* Cannot visit a doctor (s15=0)
- *Limited access:* Difficulties visiting a doctor (s15=2)
- *Full access:* Can visit a doctor easily when needed (s15 =1)

Working status

- Only the first question of the WPAI questionnaire was considered relevant to SES, as it concerns whether the participant is employed or not.

**Primary aim**

Prevalence rates with 95% CI were calculated for each DGBI and Rome subclassification group per region using the Wilson method.

**Secondary aim**

- A (fixed-effects) logistic regression analysis with dummy variables for region was applied.
- Access to health care: no and limited access to healthcare were combined into one category, given the very low numbers of participants without access to healthcare.
- Relationship status: Categories were collapsed into three groups, including “Single”, “Widowed and divorced”, and “Married and co-habiting”.
- Linearity assumption for BMI was not met. Consequently, BMI was categorized into 4 groups using the WHO definitions: underweight (BMI<18.5), normal weight (18.5-24.9), overweight (25-29.9), obese (>30).
- Linearity assumption of age was not met in the model including the esophageal DGBI group as the outcome. To account for the non-linear relationship between age and the log odds of esophageal DGBI, a restricted cubic spline with 3 degrees of freedom was applied. Model fit improved slightly after applying the spline, with the Akaike Information Criterion (AIC) decreasing from 703.86 (linear age model) to 702.64 (spline model).

**Exploratory analysis**

The influence of SES factors on DGBI group prevalence per region was investigated. Two DGBI groups (biliary disorders and central GI pain disorders) had very low prevalence, and therefore it was not worth investigating this. For the other DGBI groups, the influence of SES factors and region on the DGBI group was investigated in decreasing order of prevalence: bowel disorders, gastroduodenal disorders, esophageal disorders, and anorectal disorders. Only the models adjusting for region, SES factors, and potential confounders were investigated to reduce the number of analyses.

**Multiple testing**

All p-values including the primary analyses regarding the association between DGBIs and regions were corrected for multiple testing using the Holm-Bonferroni correction method. Corrections were not made for the secondary and the exploratory analyses.

**Missing data**

Missing data was mainly observed for BMI and SES factors (education and childhood living conditions). Missing data in other variables were likely due to skip patterns or checkbox questions and could not be classified as missing data.

**Table 1: Missingness for variables with missing data**

| **Variable** | **N = (%)** |
| --- | --- |
| Weight_valid | 171 (8.52%) |
| Height_valid | 10 (0.50%) |
| BMI_valid | 184 (9.16%) |
| Educ_valid | 21 (1.05%) |
| S10c (living conditions childhood) | 6 (0.30%) |
| RegionNL | 1 (0.05%) |
| RegionNL_Adjusted | 1 (0.05%) |

**Variables without missing data:**

Sex, Age, Commsize_binary, S5_2 (living conditions), S5_3 (living conditions), S6c (living conditions), S7 (living conditions), S8 (living conditions), S9_2 (living conditions childhood), S9_3 (living conditions childhood), S13 (access to healthcare), S14 (frequency of doctor visits), S15 (access to healthcare), S16 (access to healthcare), S40 (relationship status), Esophageal_DGBI, Gastroduodenal_DGBI, Bowel_DGBI, Anorectal_DGBI, FBilPain, CAPS, Any_DGBI, upperGIexclude, lowerGIexclude, FHrtburn, FChpain, ReflxHyp, Globus, Dysphag, FD, PDS, EPS, Belching, CNVS, CyclVom, CannHypS, FBilPain, IBS, IBStype, FConstip, OpioidIC, FDiarrh, FBloat, FBDUnspe, FecIncon, LevatAni, ProcFugx, CAPS, PHQ15sum_valid (somatic symptom scale), PHQ12sum_valid (somatic symptom scale (PHQ-15) without the 3 GI symptom questions), PHQ4sum (psychological distress, anxiety, and depression), WPAI1-6 (work productivity and activity impairment questionnaire), GlobPhys (Part of PROMIS Global 10, indicating global physical health), GlobMent (Part of PROMIS Global 10, indicating global mental health), IBS_SSS (symptom severity of IBS), ROMEIII_IBS (to assess whether Rome III IBS criteria are met), s28_1-10 (food frequency questionnaire).

**Logistic regression analysis for missingness of data**

Logistic regression analysis was done to investigate which factors were associated with missingness in BMI, education, and childhood living conditions. For each variable with missing values, the logistic regression model included all variables of interest (e.g., age, SES factors, DGBI diagnoses, questionnaires). Original variables were used instead of the computed variables to have a more detailed perspective. Variables with missing data were excluded from the model. Group DGBI variables and PHQ12sum_valid were also excluded because of multicollinearity. Results of the logistic regression analyses provided evidence against the assumption that the missing data were MCAR (i.e., missingness is completely at random). Including those variables that were associated with missingness, as identified by logistic regression, as predictors in the imputation model strengthened the plausibility of the MAR assumption.

**Table 2: Logistic regression for missingness of BMI**

| **Variable** | **OR** | **95% CI** | **P-value** |
| --- | --- | --- | --- |
| Age | *0.98* | 0.969 - 0.995 | **0.00572** |
| Sex-2 | *2.10* | 1.47 - 3.05 | **6.59e-05** |
| Commsize2 | 0.93 | 0.65 - 1.34 | 0.69702 |
| Commsize3 | 0.95 | 0.60 - 1.49 | 0.82694 |
| Commsize4 | 0.87 | 0.32 - 2.01 | 0.76573 |
| S6c | 0.94 | 0.72 - 1.19 | 0.62738 |
| S7-1 | *0.11* | 0.02 - 0.52 | **0.00474** |
| S8-1 | 0.36 | 0.03 - 9.17 | 0.43744 |
| S13-2 | 1.28 | 0.29 - 3.87 | 0.70288 |
| S13-3 | 1.18 | 0.42 - 2.75 | 0.73066 |
| S13-4 | 1.30 | 0.65 - 2.42 | 0.43446 |
| S14-2 | 0.99 | 0.49 - 2.20 | 0.97526 |
| S14-3 | 0.83 | 0.37 - 1.98 | 0.66180 |
| S14-4 | 1.09 | 0.50- 2.54 | 0.83942 |
| S14-5 | 1.21 | 0.47 - 3.24 | 0.69451 |
| S15-1 | 3.23 | 0.44 - 72.55 | 0.33329 |
| S15-2 | 2.05 | 0.23 - 49.15 | 0.57352 |
| S16-2 | 0.98 | 0.66 - 1.47 | 0.92640 |
| S16-3 | 1.63 | 0.97 - 2.73 | 0.06397 |
| S16-4 | 0.74 | 0.22 - 2.03 | 0.59475 |
| S40-2 | 0.83 | 0.55 - 1.27 | 0.40073 |
| S40-3 | 0.62 | 0.24 - 1.37 | 0.26928 |
| S40-4 | 0.57 | 0.09 - 2.05 | 0.46071 |
| S40-5 | 0.66 | 0.40 - 1.05 | 0.08831 |
| WPAI1 | 1.23 | 0.86 - 1.77 | 0.27072 |
| FHrtburn1 | *9.09* | 1.57 - 49.20 | **0.00916** |
| FChpain1 | 0.59 | 0.09 - 2.18 | 0.49405 |
| ReflxHyp1 | 0 | NA - 1.83e+31 | 0.98646 |
| Globus1 | 0 | NA - 6.07e+14 | 0.98297 |
| Dysphag1 | 0.85 | 0.18 - 2.81 | 0.80769 |
| FD1 | 0.78 | 0.28 - 1.86 | 0.59800 |
| Belching1 | 0 | NA - 2.22e+67 | 0.99121 |
| CNVS1 | 0.71 | 0.04 - 4.40 | 0.75916 |
| CyclVom1 | 2.29 | 0.10 - 19.86 | 0.50081 |
| IBS1 | 0.60 | 0.20 - 1.66 | 0.35041 |
| FConstip1 | 0.68 | 0.37 - 1.20 | 0.19950 |
| OpioidIC1 | 0.56 | 0.03 - 3.15 | 0.58931 |
| FDiarrh1 | 0.61 | 0.17 - 1.61 | 0.36859 |
| FBloat1 | 0.31 | 0.02 - 1.57 | 0.25890 |
| FBDUnspec1 | *0.49* | 0.23 - 0.94 | **0.04361** |
| FecIncon1 | 1.69 | 0.36 - 5.62 | 0.43816 |
| LevatAni1 | 2.04 | 0.37 - 8.78 | 0.36818 |
| ProcFugx1 | 0.23 | 0.01 - 1.24 | 0.17738 |
| FBilPain1 | 0 | NA - 2.71e+205 | 0.99490 |
| PHQ15sum_valid | 1.01 | 0.95 - 1.06 | 0.79219 |
| PHQ4sum | 0.98 | 0.90 - 1.08 | 0.74112 |
| GlobPhys | 0.93 | 0.86 - 1.02 | 0.11806 |
| GlobMent | 1.00 | 0.93 - 1.08 | 0.99649 |
| ROMEIII_IBS1 | 0.75 | 0.37 - 1.43 | 0.40453 |
| S28_1 | 0.95 | 0.88 - 1.03 | 0.22362 |
| S28_2 | 1.02 | 0.94 - 1.12 | 0.62970 |
| S28_3 | 0.94 | 0.80 - 1.09 | 0.40719 |
| S28_4 | 0.97 | 0.86 - 1.09 | 0.64168 |
| S28_5 | 0.99 | 0.89 - 1.10 | 0.79879 |
| S28_6 | 0.99 | 0.91 - 1.07 | 0.72963 |
| S28_7 | 1.03 | 0.95 - 1.12 | 0.50471 |
| S28_8 | 0.93 | 0.80 - 1.09 | 0.39810 |
| S28_9 | 1.04 | 0.91 - 1.20 | 0.54254 |
| S28_10 | 1.09 | 0.91 - 1.28 | 0.28652 |

**Table 3: Logistic regression for missingness of education**

| **Variable** | **OR** | **95% CI** | **P-value** |
| --- | --- | --- | --- |
| Age | 1.005 | 0.967 - 1.045 | 0.8087 |
| Sex-2 | 1.589 | 0.533 - 4.912 | 0.4081 |
| Commsize2 | 1.051 | 0.352 - 3.157 | 0.9284 |
| Commsize3 | 0.716 | 0.165 - 2.654 | 0.6296 |
| Commsize4 | 1.373 | 0.068 - 9.126 | 0.7800 |
| S6c | 0.791 | 0.344 - 1.542 | 0.5378 |
| S7-1 | *5.511e+06* | 3.398e-174 - Inf | 0.9984 |
| S8-1 | *1.329e+07* | 7.265e-316 – NA | 0.9989 |
| S13-2 | *4.492* | 0.208 - 33.117 | 0.2069 |
| S13-3 | *7.103* | 0.965 - 33.166 | **0.0230** |
| S13-4 | 1.149 | 0.057 - 7.129 | 0.9016 |
| S14-2 | 2.656 | 0.352 - 59.005 | 0.4199 |
| S14-3 | 1.971 | 0.199 - 48.224 | 0.6014 |
| S14-4 | 1.588 | 0.154 - 39.776 | 0.7258 |
| S14-5 | 2.021 | 0.061 - 70.376 | 0.6645 |
| S15-1 | *2.268e+07* | 3.368e-152 - NA | 0.9980 |
| S15-2 | *8.820e+07* | 6.091e-157 - NA | 0.9979 |
| S16-2 | 2.109 | 0.633 - 9.285 | 0.2642 |
| S16-3 | 2.485 | 0.444 - 14.366 | 0.2905 |
| S16-4 | 1.430e-07 | NA - 2.870e+78 | 0.9966 |
| S40-2 | 1.382 | 0.367 - 6.215 | 0.6481 |
| S40-3 | *6.593* | 1.285 - 35.577 | **0.0222** |
| S40-4 | 3.754e-08 | 0.000 - 2.438e+58 | 0.9962 |
| S40-5 | 1.501 | 0.265 - 7.704 | 0.6234 |
| WPAI1 | 0.493 | 0.163 - 1.440 | 0.1976 |
| FHrtburn1 | 8.837e-08 | NA - 3.910e+224 | 0.9986 |
| FChpain1 | 2.565 | 0.100 - 25.796 | 0.4769 |
| ReflxHyp1 | 1.402e-06 | NA - 6.230e+197 | 0.9987 |
| Globus1 | 9.610e-08 | NA - 5.099e+177 | 0.9982 |
| Dysphag1 | 6.736e-08 | 9.688e-44 - 1.238e+30 | 0.9959 |
| FD1 | 1.534 | 0.065 - 12.373 | 0.7308 |
| Belching1 | 2.213e-07 | 0.000 - 1.249e+284 | 0.9991 |
| CNVS1 | 2.751e-07 | NA - 2.401e+148 | 0.9980 |
| CyclVom1 | *7.300* | 2.192e-88 - 1.174e+83 | 0.9998 |
| IBS1 | 1.126e-07 | 2.167e-35 - 5.766e+21 | 0.9951 |
| FConstip1 | 1.668 | 0.326 - 6.341 | 0.4879 |
| OpioidIC1 | *15.411* | 0.614 - 168.684 | **0.0400** |
| FDiarrh1 | 2.947e-08 | NA - 3.935e+69 | 0.9955 |
| FBloat1 | 3.815e-08 | NA - 5.157e+123 | 0.9973 |
| FBDUnspec1 | 0.447 | 0.022 - 2.727 | 0.4721 |
| FecIncon1 | *5.403* | 0.255 - 41.948 | 0.1559 |
| LevatAni1 | 6.238e-07 | NA - 2.014e+134 | 0.9979 |
| ProcFugx1 | 4.205e-08 | NA - 3.777e+74 | 0.9957 |
| FBilPain1 | 2.705e-08 | NA - Inf | 0.9995 |
| PHQ15sum_valid | 0.922 | 0.759 - 1.100 | 0.3896 |
| PHQ4sum | 1.078 | 0.804 - 1.392 | 0.5849 |
| GlobPhys | 0.928 | 0.745 - 1.168 | 0.5136 |
| GlobMent | 1.110 | 0.890 - 1.390 | 0.3578 |
| ROMEIII_IBS1 | 0.650 | 0.032 - 4.392 | 0.7078 |
| S28_1 | 0.883 | 0.704 - 1.134 | 0.2975 |
| S28_2 | 1.273 | 0.940 - 1.769 | 0.1329 |
| S28_3 | 1.257 | 0.830 - 1.789 | 0.2370 |
| S28_4 | 1.056 | 0.745 - 1.437 | 0.7432 |
| S28_5 | 1.111 | 0.799 - 1.615 | 0.5549 |
| S28_6 | 1.198 | 0.929 - 1.608 | 0.1915 |
| S28_7 | 0.831 | 0.672 - 1.047 | 0.0969 |
| S28_8 | 1.440 | 0.932 - 2.189 | 0.0928 |
| S28_9 | 0.939 | 0.606 - 1.371 | 0.7590 |
| S28_10 | 0.648 | 0.140 - 1.210 | 0.3711 |

**Table 4: Logistic regression for missingness of childhood living conditions**

| **Variable** | **OR** | **95% CI** | **P-value** |
| --- | --- | --- | --- |
| Age | *1.015* | 1.000 - 1.030 | **0.04516** |
| Sex-2 | 0.859 | 0.578 - 1.273 | 0.45089 |
| Commsize2 | 1.259 | 0.831 - 1.916 | 0.27966 |
| Commsize3 | *1.791* | 1.072 - 2.965 | **0.02427** |
| Commsize4 | *2.082* | 0.970 - 4.198 | **0.04808** |
| S6c | 0.999 | 0.745 - 1.307 | 0.99681 |
| S7-1 | 0.722 | 0.087 - 18.666 | 0.79924 |
| S8-1 | *5.47e+06* | 3.85e-44 - NA | 0.98855 |
| S13-2 | *4.265* | 1.484 - 10.666 | **0.00340** |
| S13-3 | *2.783* | 1.128 - 6.214 | **0.01766** |
| S13-4 | *2.124* | 1.041 - 4.067 | **0.02934** |
| S14-2 | 1.549 | 0.723 - 3.696 | 0.28864 |
| S14-3 | 1.128 | 0.470 - 2.925 | 0.79406 |
| S14-4 | 1.144 | 0.490 - 2.919 | 0.76516 |
| S14-5 | 2.430 | 0.893 - 6.968 | 0.08794 |
| S15-1 | 0.233 | 0.055 - 1.112 | 0.05323 |
| S15-2 | 0.362 | 0.074 - 1.921 | 0.21520 |
| S16-2 | 0.694 | 0.468 - 1.036 | 0.07073 |
| S16-3 | 0.784 | 0.420 - 1.415 | 0.43098 |
| S16-4 | 1.608 | 0.599 - 3.884 | 0.31458 |
| S40-2 | 1.198 | 0.740 - 1.962 | 0.46759 |
| S40-3 | 0.926 | 0.359 - 2.130 | 0.86387 |
| S40-4 | *3.146* | 1.253 - 7.461 | **0.01123** |
| S40-5 | 0.720 | 0.373 - 1.329 | 0.30714 |
| WPAI1 | 1.144 | 0.764 - 1.720 | 0.51530 |
| FHrtburn1 | 3.288 | 0.413 - 17.794 | 0.19591 |
| FChpain1 | 0.394 | 0.021 - 2.011 | 0.37292 |
| ReflxHyp1 | 1.154 | 0.050 - 10.364 | 0.90903 |
| Globus1 | 1.34e-06 | NA - 1.31e+15 | 0.98386 |
| Dysphag1 | 1.168 | 0.372 - 3.211 | 0.77474 |
| FD1 | 1.428 | 0.580 - 3.244 | 0.41440 |
| Belching1 | 1.09e-06 | NA - 2.46e+65 | 0.99205 |
| CNVS1 | 2.352 | 0.333 - 11.325 | 0.32946 |
| CyclVom1 | 1.490 | 0.112 - 11.731 | 0.73119 |
| IBS1 | 0.529 | 0.160 - 1.515 | 0.26010 |
| FConstip1 | 0.670 | 0.313 - 1.315 | 0.27039 |
| OpioidIC1 | 1.687 | 0.323 - 6.603 | 0.48632 |
| FDiarrh1 | 0.370 | 0.056 - 1.358 | 0.20137 |
| FBloat1 | 1.536 | 0.329 - 5.063 | 0.52623 |
| FBDUnspec1 | 0.454 | 0.181 - 0.991 | 0.06560 |
| FecIncon1 | 0.385 | 0.045 - 1.818 | 0.29756 |
| LevatAni1 | 1.581 | 0.221 - 6.982 | 0.58648 |
| ProcFugx1 | 2.199 | 0.691 - 6.051 | 0.14936 |
| FBilPain1 | 1.13e-06 | NA - 1.51e+206 | 0.99545 |
| PHQ15sum_valid | *1.087* | 1.022 - 1.155 | **0.00766** |
| PHQ4sum | 1.103 | 0.997 - 1.217 | 0.05432 |
| GlobPhys | *1.135* | 1.028 - 1.256 | **0.01293** |
| GlobMent | 1.030 | 0.949 - 1.120 | 0.47904 |
| ROMEIII_IBS1 | 0.913 | 0.437 - 1.800 | 0.80088 |
| S28_1 | 0.932 | 0.852 - 1.023 | 0.13325 |
| S28_2 | 1.065 | 0.964 - 1.178 | 0.22007 |
| S28_3 | 0.994 | 0.846 - 1.158 | 0.94221 |
| S28_4 | 1.055 | 0.932 - 1.189 | 0.38615 |
| S28_5 | 0.909 | 0.809 - 1.023 | 0.10886 |
| S28_6 | *1.110* | 1.010 - 1.226 | **0.03390** |
| S28_7 | 0.979 | 0.897 - 1.074 | 0.64829 |
| S28_8 | 0.873 | 0.732 - 1.036 | 0.12543 |
| S28_9 | 1.107 | 0.945 - 1.285 | 0.19334 |
| S28_10 | *1.218* | 1.048 - 1.397 | **0.00655** |

**Multiple imputation**

The three variables of interest with missing data (bmi_valid, educ_valid, s10c) had a total percentage of missingness of 10.51%. These variables were imputed in the model. The predictors included outcomes (DGBIs, but only the DGBI groups with a prevalence >1% were included in the model), independent variables (SES factors, age, sex), and factors that were significant in the logistic regression analysis for missingness (s16, s13, PHQ4sum, PHQ15sum_valid, GlobPhys, s28_5, s28_6, s28_7, s28_10). No interactions were included in the imputation model. Predictive mean matching was the method used for imputation because the variables with missing data were all numerical. The number of imputed datasets was m = 15. Convergence was checked graphically suggesting that 30 iterations were sufficient.

Predictors that were included in the model: Any_DGBI, esophageal_DGBI, gastroduodenal_DGBI, bowel_DGBI, anorectal_DGBI, RegionNL_Adjusted, age, sex, commsize_binary, LivingConditions_Adjusted, LivingConditions_Childhood_Adjusted, Healthcare_Access_Adjusted, WPAI1, relationship_status, s16, s13, PHQ4sum, PHQ15sum_valid, GlobPhys, s28_5, s28_6, s28_7, s28_10.

**Logistic regression on imputed datasets**

The influence of SES factors on DGBI prevalence was investigated on the imputed datasets. The three same models were applied as for the complete case analysis, yielding similar results. Model assumptions (e.g., linearity, no influential outliers, no multicollinearity) were checked randomly for 3 imputed datasets (1, 5, and 9). Linearity was violated for BMI, so BMI was categorized in four groups according to the WHO definitions. No other violations were detected. Model fit (AIC & BIC) was reported for every imputed dataset.

**2.Results**

**2.1 Additional results from multiple imputation analyses**

**2.1.1 Socio-economic influence on any DGBI prevalence**

**Table 5: Model 1** - A crude model including only region to show unadjusted association between region and DGBI prevalence.

|  | **OR** | **95% CI** | **P-value** |
| --- | --- | --- | --- |
| Region 2 | 0.99 | 0.78 – 1.26 | 0.934 |
| Region 3 | 1.05 | 0.82 – 1.35 | 0.702 |

These findings are based on multiple imputation analysis. Reference: Region 1. AIC=2479. BIC = 2496. *OR, Odds Ratio; 95% CI, 95% confidence interval.*

**Table 6: Model 2 -** A model including region and socio-economic status to assess the effect of socio-economic factors.

|  | **OR** | **95% CI** | **P-value** |
| --- | --- | --- | --- |
| Region 2 | 0.95 | 0.75 – 1.22 | 0.707 |
| Region 3 | 1.08 | 0.84 – 1.40 | 0.542 |
| Education (years) | 1.03 | 1.00 – 1.05 | **0.026** |
| Community size  *Up to 50,000 inhabitants* | 0.90 | 0.73 – 1.10 | 0.286 |
| Current living conditions  *Good*  *Poor* | 0.95  1.20 | 0.77 – 1.17  0.70 – 2.07 | 0.627  0.509 |
| Childhood living conditions  *Good*  *Poor* | 1.42  0.87 | 1.14 – 1.76  0.57 – 1.31 | **0.002**  0.491 |
| Healthcare Access  *Limited/No Access* | 2.72 | 1.81 – 4.08 | **<0.0001** |
| Work status  *Employed* | 0.73 | 0.60 – 0.89 | **0.003** |
| Relationship status  *Divorced/Widowed*  *Married/co-habiting* | 1.19  0.82 | 0.81 – 1.73  0.66 – 1.02 | 0.380  0.075 |

These findings are based on multiple imputation analysis. References: Region (Region1), Community size (50,000 and above inhabitants), Current living conditions (Average), Childhood living conditions (Average), Healthcare access (Full access), Work status (unemployed), Relationship status. *OR, Odds Ratio; 95% CI, 95% confidence interval.*

**Table 7: Model 3 -** A fully adjusted model including region, socio-economic status, and age, sex, and BMI as potential confounders.

|  | **OR** | **95% CI** | **P-value** |
| --- | --- | --- | --- |
| Region 2 | 0.89 | 0.69 – 1.14 | 0.352 |
| Region 3 | 1.02 | 0.78 – 1.33 | 0.872 |
| Education (years) | 1.02 | 1.00 – 1.05 | 0.068 |
| Community size  *Up to 50,000 inhabitants* | 0.85 | 0.69 – 1.05 | 0.123 |
| Current living conditions  *Good*  *Poor* | 1.00  1.18 | 0.81 – 1.24  0.67 – 2.09 | 0.975  0.563 |
| Childhood living conditions  *Good*  *Poor* | 1.09  0.86 | 0.86 – 1.39  0.56 – 1.32 | 0.461  0.495 |
| Healthcare Access  *Limited/No Access* | 2.47 | 1.62 – 3.77 | **<0.0001** |
| Work status  *Employed* | 0.65 | 0.52 – 0.81 | **<0.001** |
| Relationship status  *Divorced/Widowed*  *Married/co-habiting* | 1.50  1.04 | 1.00 – 2.26  0.82 – 1.33 | 0.051  0.735 |
| Age | 0.98 | 0.98 – 0.99 | **<0.0001** |
| Sex  *Female* | 2.10 | 1.70 – 2.59 | **<0.0001** |
| BMI  *Underweight*  *Overweight*  *Obese* | 2.53  0.95  1.59 | 1.36 – 4.73  0.74 – 1.22  1.19 – 2.13 | **0.004**  0.692  **0.002** |

These findings are based on multiple imputation analysis. References: Region (Region1), Community size (50,000 and above inhabitants), Current living conditions (Average), Childhood living conditions (Average), Healthcare access (Full access), Work status (unemployed), Relationship status (Single), Sex (Male), BMI (Normal weight). BMI was categorized based on the WHO categories. *OR, Odds Ratio; 95% CI, 95% confidence interval.*

**Table 8: AIC Model 2**

| **Imputation dataset** | **AIC** |
| --- | --- |
| Imp1 | 2440.618 |
| Imp2 | 2441.052 |
| Imp3 | 2440.696 |
| Imp4 | 2440.631 |
| Imp5 | 2440.541 |
| Imp6 | 2440.588 |
| Imp7 | 2440.665 |
| Imp8 | 2440.209 |
| Imp9 | 2441.256 |
| Imp10 | 2439.859 |
| Imp11 | 2439.897 |
| Imp12 | 2439.859 |
| Imp13 | 2440.391 |
| Imp14 | 2440.617 |
| Imp15 | 2440.115 |

*AIC, Akaike Information Criterion.*

**Table 9: BIC Model 2**

| **Imputation dataset** | **BIC** |
| --- | --- |
| Imp1 | 2513.475 |
| Imp2 | 2513.909 |
| Imp3 | 2513.554 |
| Imp4 | 2513.488 |
| Imp5 | 2513.398 |
| Imp6 | 2513.445 |
| Imp7 | 2513.522 |
| Imp8 | 2513.066 |
| Imp9 | 2514.113 |
| Imp10 | 2512.716 |
| Imp11 | 2512.754 |
| Imp12 | 2512.716 |
| Imp13 | 2513.248 |
| Imp14 | 2513.475 |
| Imp15 | 2512.972 |

*BIC, Bayesian Information Criterion*

**Table 10: AIC Model 3**

| **Imputation dataset** | **AIC** |
| --- | --- |
| Imp1 | 2342.240 |
| Imp2 | 2335.273 |
| Imp3 | 2337.490 |
| Imp4 | 2335.331 |
| Imp5 | 2336.640 |
| Imp6 | 2338.522 |
| Imp7 | 2340.836 |
| Imp8 | 2336.886 |
| Imp9 | 2331.361 |
| Imp10 | 2336.665 |
| Imp11 | 2336.629 |
| Imp12 | 2338.035 |
| Imp13 | 2338.243 |
| Imp14 | 2335.393 |
| Imp15 | 2337.998 |

*AIC, Akaike Information Criterion.*

**Table 11: BIC Model 3**

| **Imputation dataset** | **BIC** |
| --- | --- |
| Imp1 | 2443.119 |
| Imp2 | 2436.152 |
| Imp3 | 2438.369 |
| Imp4 | 2436.210 |
| Imp5 | 2437.519 |
| Imp6 | 2439.401 |
| Imp7 | 2441.715 |
| Imp8 | 2437.765 |
| Imp9 | 2432.240 |
| Imp10 | 2437.544 |
| Imp11 | 2437.509 |
| Imp12 | 2438.914 |
| Imp13 | 2439.122 |
| Imp14 | 2436.272 |
| Imp15 | 2438.877 |

*BIC, Bayesian Information Criterion*

**Table 12:** Likelihood Ratio Tests

| **Comparison** | **Test statistic (D3)** | **df (Δ)** | **P-value** |
| --- | --- | --- | --- |
| Model 1 vs. Model 2 | 5.87 | 10 | **<0.0001** |
| Model 2 vs. Model 3 | 21.05 | 5 | **<0.0001** |

**Table 13:** ROC/AUROC for model 3 selected based on AIC and BIC

| **Imputation dataset** | **AUROC** | **95% CI** |
| --- | --- | --- |
| Imp 1 | 0.6754772 | 0.6502376 - 0.7007168 |
| Imp2 | 0.6777714 | 0.6525707 - 0.7029720 |
| Imp3 | 0.6763836 | 0.6512045 - 0.7015628 |
| Imp4 | 0.6772323 | 0.6520363 - 0.7024282 |
| Imp5 | 0.6768351 | 0.6516660 - 0.7020042 |
| Imp6 | 0.6765407 | 0.6513576 - 0.7017239 |
| Imp7 | 0.6756979 | 0.6504601 - 0.7009358 |
| Imp8 | 0.6780657 | 0.6528439 - 0.7032876 |
| Imp9 | 0.6784459 | 0.6532991 - 0.7035927 |
| Imp10 | 0.6774051 | 0.6521912 - 0.7026191 |
| Imp11 | 0.6773170 | 0.6520973 - 0.7025366 |
| Imp12 | 0.6761266 | 0.6509550 - 0.7012983 |
| Imp13 | 0.6767691 | 0.6515344 - 0.7020038 |
| Imp14 | 0.6780272 | 0.6527861 - 0.7032683 |
| Imp15 | 0.6779717 | 0.6528824 - 0.7030610 |

Median AUROC = 0.68. *ROC, Receiver Operating Characteristic; AUROC, Area Under the Receiver Operating Characteristic curve.*

**2.1.2 Socio-economic influence on DGBI group prevalence**

**Bowel DGBI**

**Table 14:** A fully adjusted model for bowel DGBI including region, socio-economic status, and age, sex, and BMI as potential confounders.

|  | **OR** | **95% CI** | **P-value** |
| --- | --- | --- | --- |
| Region 2 | 0.94 | 0.72 – 1.22 | 0.635 |
| Region 3 | 1.00 | 0.76 – 1.32 | 0.988 |
| Education (years) | 1.03 | 1.00 – 1.05 | **0.041** |
| Community size  *Up to 50,000 inhabitants* | 0.83 | 0.66 – 1.03 | 0.086 |
| Current living conditions  *Good*  *Poor* | 0.97  1.32 | 0.78 – 1.22  0.73 – 2.38 | 0.823  0.361 |
| Childhood living conditions  *Good*  *Poor* | 1.07  0.67 | 0.83 – 1.37  0.42 – 1.09 | 0.592  0.104 |
| Healthcare Access  *Limited/No Access* | 2.10 | 1.37 – 3.21 | **<0.001** |
| Work status  *Employed* | 0.68 | 0.54 – 0.86 | **0.001** |
| Relationship status  *Divorced/Widowed*  *Married/co-habiting* | 1.36  1.03 | 0.88 – 2.09  0.80 – 1.33 | 0.162  0.808 |
| Age | 0.98 | 0.97 – 0.99 | **<0.0001** |
| Sex  *Female* | 2.19 | 1.75 – 2.72 | **<0.0001** |
| BMI  *Underweight*  *Overweight*  *Obese* | 2.03  0.91  1.56 | 1.10 – 3.73  0.70 – 1.20  1.16 – 2.11 | **0.023**  0.514  **0.004** |

These findings are based on multiple imputation analysis. References: Region (Region1), Community size (50,000 and above inhabitants), Current living conditions (Average), Childhood living conditions (Average), Healthcare access (Full access), Work status (unemployed), Relationship status (Single), Sex (Male), BMI (Normal weight). BMI was categorized based on the WHO categories. *OR, Odds Ratio; 95% CI, 95% confidence interval; BMI, body mass index.*

**Gastroduodenal DGBI**

**Table 15:** A fully adjusted model for gastroduodenal DGBI including region, socio-economic status, and age, sex, and BMI as potential confounders.

|  | **OR** | **95% CI** | **P-value** |
| --- | --- | --- | --- |
| Region 2 | 1.34 | 0.80 – 2.25 | 0.272 |
| Region 3 | 1.58 | 0.94 – 2.67 | 0.087 |
| Education (years) | 1.03 | 0.98 – 1.08 | 0.238 |
| Community size  *Up to 50,000 inhabitants* | 1.10 | 0.73 – 1.64 | 0.654 |
| Current living conditions  *Good*  *Poor* | 0.90  1.25 | 0.59 – 1.35  0.50 – 3.15 | 0.597  0.635 |
| Childhood living conditions  *Good*  *Poor* | 0.82  1.75 | 0.51 – 1.30  0.88 – 3.48 | 0.390  0.111 |
| Healthcare Access  *Limited/No Access* | 1.97 | 1.04 – 3.72 | **0.037** |
| Work status  *Employed* | 0.62 | 0.41 – 0.93 | **0.022** |
| Relationship status  *Divorced/Widowed*  *Married/co-habiting* | 1.50  1.07 | 0.70 – 3.21  0.68 – 1.68 | 0.301  0.785 |
| Age | 0.98 | 0.96 – 0.99 | **0.002** |
| Sex  *Female* | 1.27 | 0.85 – 1.89 | 0.242 |
| BMI  *Underweight*  *Overweight*  *Obese* | 5.09  0.96  1.69 | 2.48 – 10.43  0.58 – 1.58  0.99 – 2.88 | **<0.0001**  0.818  0.055 |

These findings are based on multiple imputation analysis. References: Region (Region1), Community size (50,000 and above inhabitants), Current living conditions (Average), Childhood living conditions (Average), Healthcare access (Full access), Work status (unemployed), Relationship status (Single), Sex (Male), BMI (Normal weight). BMI was categorized based on the WHO categories. *OR, Odds Ratio; 95% CI, 95% confidence interval; BMI, body mass index.*

**Esophageal DGBI**

**Table 16:** A fully adjusted model for esophageal DGBI including region, socio-economic status, and age, sex, and BMI as potential confounders.

|  | **OR** | **95% CI** | **P-value** |
| --- | --- | --- | --- |
| Region 2 | 0.72 | 0.43 – 1.23 | 0.227 |
| Region 3 | 0.97 | 0.57 – 1.65 | 0.916 |
| Education (years) | 0.97 | 0.92 – 1.02 | 0.186 |
| Community size  *Up to 50,000 inhabitants* | 0.93 | 0.59 – 1.44 | 0.732 |
| Current living conditions  *Good*  *Poor* | 0.93  1.17 | 0.59 – 1.46  0.38 – 3.56 | 0.749  0.786 |
| Childhood living conditions  *Good*  *Poor* | 1.14  1.07 | 0.68 – 1.92  0.47 – 2.45 | 0.613  0.875 |
| Healthcare Access  *Limited/No Access* | 1.60 | 0.76 – 3.37 | 0.218 |
| Work status  *Employed* | 0.56 | 0.35 – 0.89 | **0.015** |
| Relationship status  *Divorced/Widowed*  *Married/co-habiting* | 1.88  1.19 | 0.86 – 4.10  0.70 – 2.03 | 0.112  0.519 |
| Age | 0.99 | 0.98 – 1.01 | 0.381 |
| Sex  *Female* | 1.44 | 0.92 – 2.25 | 0.108 |
| BMI  *Underweight*  *Overweight*  *Obese* | 1.56  1.18  1.08 | 0.51 – 4.77  0.70 – 2.00  0.57 – 2.05 | 0.435  0.535  0.805 |

These findings are based on multiple imputation analysis. References: Region (Region1), Community size (50,000 and above inhabitants), Current living conditions (Average), Childhood living conditions (Average), Healthcare access (Full access), Work status (unemployed), Relationship status (Single), Sex (Male), BMI (Normal weight). BMI was categorized based on the WHO categories. *OR, Odds Ratio; 95% CI, 95% confidence interval; BMI, body mass index.*

**Anorectal DGBI**

**Table 17:** A fully adjusted model for anorectal DGBI including region, socio-economic status, and age, sex, and BMI as potential confounders.

|  | **OR** | **95% CI** | **P-value** |
| --- | --- | --- | --- |
| Region 2 | 0.69 | 0.39 – 1.21 | 0.194 |
| Region 3 | 1.26 | 0.73 – 2.16 | 0.411 |
| Education (years) | 1.03 | 0.98 – 1.09 | 0.276 |
| Community size  *Up to 50,000 inhabitants* | 0.56 | 0.36 – 0.88 | **0.012** |
| Current living conditions  *Good*  *Poor* | 1.55  1.15 | 0.95 – 2.53  0.33 – 3.98 | 0.078  0.829 |
| Childhood living conditions  *Good*  *Poor* | 1.13  1.18 | 0.67 – 1.93  0.52 – 2.70 | 0.647  0.692 |
| Healthcare Access  *Limited/No Access* | 3.31 | 1.70 – 6.44 | **<0.001** |
| Work status  *Employed* | 0.86 | 0.52 – 1.40 | 0.543 |
| Relationship status  *Divorced/Widowed*  *Married/co-habiting* | 1.97  1.06 | 0.89 – 4.37  0.60 – 1.86 | 0.096  0.834 |
| Age | 1.00 | 0.99 – 1.02 | 0.594 |
| Sex  *Female* | 1.47 | 0.93 – 2.32 | 0.099 |
| BMI  *Underweight*  *Overweight*  *Obese* | 2.97  1.26  2.08 | 1.10 – 7.98  0.71 – 2.23  1.15 – 3.78 | **0.031**  0.427  **0.016** |

These findings are based on multiple imputation analysis. References: Region (Region1), Community size (50,000 and above inhabitants), Current living conditions (Average), Childhood living conditions (Average), Healthcare access (Full access), Work status (unemployed), Relationship status (Single), Sex (Male), BMI (Normal weight). BMI was categorized based on the WHO categories. *OR, Odds Ratio; 95% CI, 95% confidence interval; BMI, body mass index.*

**2.2 Results from complete case analyses**

**2.2.1** **Socio-economic influence on any DGBI prevalence**

**Table 18: Model 1** - A crude model including only region to show unadjusted association between region and DGBI prevalence.

|  | **OR** | **95% CI** | **P-value** |
| --- | --- | --- | --- |
| Region 2 | 0.99 | 0.78 – 1.26 | 0.934 |
| Region 3 | 1.05 | 0.82 – 1.35 | 0.702 |

These findings are based on complete case analyses. Reference: Region 1. AIC = 2479.187. BIC = 2496. *OR, Odds Ratio; 95% CI, 95% confidence interval.*

**Table 19: Model 2 -** A model including region and socio-economic status to assess the effect of socio-economic factors.

|  | **OR** | **95% CI** | **P-value** |
| --- | --- | --- | --- |
| Region 2 | 0.96 | 0.75 – 1.23 | 0.719 |
| Region 3 | 1.09 | 0.84 – 1.41 | 0.504 |
| Education (years) | 1.03 | 1.00 – 1.05 | **0.027** |
| Community size  *Up to 50,000 inhabitants* | 0.90 | 0.73 – 1.10 | 0.313 |
| Current living conditions  *Good*  *Poor* | 0.94  1.19 | 0.76 – 1.16  0.68 – 2.06 | 0.572  0.533 |
| Childhood living conditions  *Good*  *Poor* | 1.41  0.83 | 1.14 – 1.76  0.54 – 1.25 | **0.002**  0.386 |
| Healthcare Access  *Limited/No Access* | 2.71 | 1.80 – 4.10 | **<0.0001** |
| Work status  *Employed* | 0.74 | 0.60 – 0.91 | **0.004** |
| Relationship status  *Divorced/Widowed*  *Married/co-habiting* | 1.26  0.83 | 0.86 – 1.84  0.67 – 1.04 | 0.238  0.104 |

These findings are based on complete case analyses. References: Region (Region1), Community size (50,000 and above inhabitants), Current living conditions (Average), Childhood living conditions (Average), Healthcare access (Full access), Work status (unemployed), Relationship status (Single). AIC = 2416.199. BIC = 2488.919. *OR, Odds Ratio; 95% CI, 95% confidence interval.*

**Table 20: Model 3 -** A fully adjusted model including region, socio-economic status, and age, sex, and BMI as potential confounders.

|  | **OR** | **95% CI** | **P-value** |
| --- | --- | --- | --- |
| Region 2 | 0.88 | 0.68 – 1.16 | 0.369 |
| Region 3 | 1.01 | 0.76 – 1.33 | 0.953 |
| Education (years) | 1.03 | 1.00 – 1.06 | **0.030** |
| Community size  *Up to 50,000 inhabitants* | 0.79 | 0.63 – 0.99 | **0.039** |
| Current living conditions  *Good*  *Poor* | 1.01  1.07 | 0.81 – 1.27  0.57 – 1.96 | 0.910  0.841 |
| Childhood living conditions  *Good*  *Poor* | 1.10  0.83 | 0.85 – 1.41  0.52 – 1.30 | 0.480  0.434 |
| Healthcare Access  *Limited/No Access* | 2.68 | 1.72 – 4.21 | **<0.0001** |
| Work status  *Employed* | 0.64 | 0.50 – 0.81 | **<0.001** |
| Relationship status  *Divorced/Widowed*  *Married/co-habiting* | 1.55 | 1.00 – 2.38  0.82 – 1.38 | **0.048**  0.640 |
| Age | 0.98 | 0.97 – 0.99 | **<0.0001** |
| Sex  *Female* | 2.19 | 1.76 – 2.72 | **<0.0001** |
| BMI  *Underweight*  *Overweight*  *Obese* | 2.76  0.93  1.63 | 1.50 – 5.20  0.72 – 1.19  1.21 – 2.19 | **0.001**  0.553  **0.001** |

These findings are based on complete case analyses. References: Region (Region1), Community size (50,000 and above inhabitants), Current living conditions (Average), Childhood living conditions (Average), Healthcare access (Full access), Work status (unemployed), Relationship status (Single), Sex (Male), BMI (Normal weight). BMI was categorized based on the WHO categories. AIC = 2098.064. BIC = 2197.044. *OR, Odds Ratio; 95% CI, 95% confidence interval; BMI, body mass index.*

**Table 21:** Likelihood Ratio Tests

| **Comparison** | **df (Δ)** | **χ²** | **P-value** |
| --- | --- | --- | --- |
| Model 1 vs. Model 2 | 10 | 61.77 | **<0.0001** |
| Model 2 vs. Model 3 | 5 | 115.66 | **<0.0001** |

**Table 22:** ROC/AUROC for model 3 selected based on AIC and BIC

| **Model** | **AUROC** | **95% CI** |
| --- | --- | --- |
| Model 3 | 0.687 | 0.66 – 0.71 |

*ROC, Receiver Operating Characteristic; AUROC, Area Under the Receiver Operating Characteristic curve.*

**2.2.2 Socio-economic influence on DGBI group prevalence**

**Bowel DGBI**

**Table 23:** A fully adjusted model for bowel DGBI including region, socio-economic status, and age, sex, and BMI as potential confounders.

|  | **OR** | **95% CI** | **P-value** |
| --- | --- | --- | --- |
| Region 2 | 0.96 | 0.72 – 1.27 | 0.765 |
| Region 3 | 1.02 | 0.76 – 1.37 | 0.905 |
| Education (years) | 1.03 | 1.01 – 1.06 | **0.019** |
| Community size  *Up to 50,000 inhabitants* | 0.78 | 0.62 – 0.99 | **0.039** |
| Current living conditions  *Good*  *Poor* | 0.98  1.15 | 0.77 – 1.24  0.59 – 2.18 | 0.865  0.666 |
| Childhood living conditions  *Good*  *Poor* | 1.05  0.64 | 0.80 – 1.36  0.38 – 1.05 | 0.733  0.085 |
| Healthcare Access  *Limited/No Access* | 2.27 | 1.45 – 3.56 | **<0.001** |
| Work status  *Employed* | 0.68 | 0.53 – 0.87 | **0.002** |
| Relationship status  *Divorced/Widowed*  *Married/co-habiting* | 1.45  1.05 | 0.92 - 2.27  0.81 – 1.38 | 0.108  0.702 |
| Age | 0.98 | 0.97 – 0.99 | **<0.0001** |
| Sex  *Female* | 2.33 | 1.85 – 2.94 | **<0.0001** |
| BMI  *Underweight*  *Overweight*  *Obese* | 2.15  0.88  1.59 | 1.18 – 3.96  0.67 – 1.15  1.17 – 2.15 | **0.013**  0.339  **0.003** |

These findings are based on complete case analyses. References: Region (Region1), Community size (50,000 and above inhabitants), Current living conditions (Average), Childhood living conditions (Average), Healthcare access (Full access), Work status (unemployed), Relationship status (Single), Sex (Male), BMI (Normal weight). BMI was categorized based on the WHO categories. AIC = 1963.279. BIC = 2062.258. *OR, Odds Ratio; 95% CI, 95% confidence interval; BMI, body mass index.*

**Gastroduodenal DGBI**

**Table 24:** A fully adjusted model for gastroduodenal DGBI including region, socio-economic status, and age, sex, and BMI as potential confounders.

|  | **OR** | **95% CI** | **P-value** |
| --- | --- | --- | --- |
| Region 2 | 1.62 | 0.94 – 2.90 | 0.093 |
| Region 3 | 1.73 | 0.99 – 3.13 | 0.060 |
| Education (years) | 1.02 | 0.97 – 1.07 | 0.403 |
| Community size  *Up to 50,000 inhabitants* | 1.09 | 0.71 – 1.67 | 0.708 |
| Current living conditions  *Good*  *Poor* | 0.91  1.45 | 0.59 – 1.42  0.53 – 3.66 | 0.689  0.454 |
| Childhood living conditions  *Good*  *Poor* | 0.82  1.71 | 0.50 – 1.33  0.79 – 3.43 | 0.422  0.152 |
| Healthcare Access  *Limited/No Access* | 2.19 | 1.10 – 4.11 | **0.019** |
| Work status  *Employed* | 0.62 | 0.40 – 0.96 | **0.031** |
| Relationship status  *Divorced/Widowed*  *Married/co-habiting* | 1.46  1.06 | 0.62 – 3.20  0.66 – 1.72 | 0.359  0.816 |
| Age | 0.98 | 0.96 – 0.99 | **0.003** |
| Sex  *Female* | 1.23 | 0.81 – 1.87 | 0.336 |
| BMI  *Underweight*  *Overweight*  *Obese* | 5.41  0.91  1.72 | 2.57 – 11.01  0.54 – 1.52  0.99 – 2.94 | **<0.0001**  0.727  **0.049** |

These findings are based on complete case analyses. References: Region (Region1), Community size (50,000 and above inhabitants), Current living conditions (Average), Childhood living conditions (Average), Healthcare access (Full access), Work status (unemployed), Relationship status (Single), Sex (Male), BMI (Normal weight). BMI was categorized based on the WHO categories. AIC = 796.1058. BIC = 895.0855. *OR, Odds Ratio; 95% CI, 95% confidence interval; BMI, body mass index.*

**Esophageal DGBI**

**Table 25:** A fully adjusted model for esophageal DGBI including region, socio-economic status, and age, sex, and BMI as potential confounders.

|  | **OR** | **95% CI** | **P-value** |
| --- | --- | --- | --- |
| Region 2 | 0.82 | 0.47 – 1.44 | 0.481 |
| Region 3 | 0.94 | 0.53 – 1.67 | 0.830 |
| Education (years) | 0.95 | 0.90 – 1.00 | 0.055 |
| Community size  *Up to 50,000 inhabitants* | 0.89 | 0.56 – 1.42 | 0.610 |
| Current living conditions  *Good*  *Poor* | 1.01  1.37 | 0.63 – 1.65  0.39 – 4.07 | 0.953  0.589 |
| Childhood living conditions  *Good*  *Poor* | 1.15  1.03 | 0.66 – 1.98  0.39 – 2.40 | 0.621  0.941 |
| Healthcare Access  *Limited/No Access* | 1.45 | 0.60 – 3.05 | 0.368 |
| Work status  *Employed* | 0.49 | 0.29 – 0.82 | **0.006** |
| Relationship status  *Divorced/Widowed*  *Married/co-habiting* | 1.38  0.96 | 0.58 – 3.09  0.55 – 1.68 | 0.441  0.874 |
| Age  Age1  Age2  Age3 | 1.48  0.89  0.17 | 0.45 – 5.04  0.07 – 10.94  0.02 – 1.07 | 0.523  0.929  0.079 |
| Sex  *Female* | 1.26 | 0.79 – 2.02 | 0.326 |
| BMI  *Underweight*  *Overweight*  *Obese* | 1.77  1.14  0.99 | 0.50 – 4.93  0.67 – 1.92  0.51 – 1.84 | 0.315  0.632  0.976 |

These findings are based on complete case analyses. References: Region (Region1), Community size (50,000 and above inhabitants), Current living conditions (Average), Childhood living conditions (Average), Healthcare access (Full access), Work status (unemployed), Relationship status (Single), Sex (Male), BMI (Normal weight). BMI was categorized based on the WHO categories. Linearity assumption for age was violated (p=0.031), which was modeled using restricted cubic spline. AIC = 702.6375. BIC = 812.6149. *OR, Odds Ratio; 95% CI, 95% confidence interval; BMI, body mass index.*

**Anorectal DGBI**

**Table 26:** A fully adjusted model for anorectal DGBI including region, socio-economic status, and age, sex, and BMI as potential confounders.

|  | **OR** | **95% CI** | **P-value** |
| --- | --- | --- | --- |
| Region 2 | 0.59 | 0.32 – 1.08 | 0.082 |
| Region 3 | 1.29 | 0.74 – 2.28 | 0.375 |
| Education (years) | 1.03 | 0.97 – 1.09 | 0.298 |
| Community size  *Up to 50,000 inhabitants* | 0.49 | 0.30 – 0.79 | **0.003** |
| Current living conditions  *Good*  *Poor* | 1.62  1.50 | 0.97 – 2.76  0.37 – 4.94 | 0.068  0.532 |
| Childhood living conditions  *Good*  *Poor* | 1.24  0.89 | 0.70 – 2.14  0.32 – 2.13 | 0.452  0.813 |
| Healthcare Access  *Limited/No Access* | 3.40 | 1.62 – 6.63 | **0.001** |
| Work status  *Employed* | 1.00 | 0.59 – 1.70 | 0.988 |
| Relationship status  *Divorced/Widowed*  *Married/co-habiting* | 2.13  1.14 | 0.89 – 4.93  0.63 – 2.13 | 0.079  0.666 |
| Age | 1.01 | 0.99 – 1.03 | 0.468 |
| Sex  *Female* | 1.45 | 0.90 – 2.34 | 0.126 |
| BMI  *Underweight*  *Overweight*  *Obese* | 3.46  1.35  2.31 | 1.17 – 8.89  0.76 – 2.40  1.24 – 4.26 | **0.015**  0.310  **0.007** |

These findings are based on complete case analyses. References: Region (Region1), Community size (50,000 and above inhabitants), Current living conditions (Average), Childhood living conditions (Average), Healthcare access (Full access), Work status (unemployed), Relationship status (Single), Sex (Male), BMI (Normal weight). BMI was categorized based on the WHO categories. AIC = 653.0785. BIC = 752.0582. *OR, Odds Ratio; 95% CI, 95% confidence interval; BMI, body mass index.*
